# On the Absolutely Continuous Spectrum of Generalized Indefinite Strings

**DOI:** 10.1007/s00023-021-01072-x

**Published:** 2021-06-11

**Authors:** Jonathan Eckhardt, Aleksey Kostenko

**Affiliations:** 1 Department of Mathematical Sciences, Loughborough University, Epinal Way, Loughborough, Leicestershire LE11 3TU UK; 2 Faculty of Mathematics, University of Vienna, Oskar-Morgenstern-Platz 1, 1090 Wien, Austria; 3 Faculty of Mathematics and Physics, University of Ljubljana, Jadranska 19, 1000 Ljubljana, Slovenia

**Keywords:** Primary 34L05, 34B07, Secondary 34L25, 37K15

## Abstract

We investigate absolutely continuous spectrum of generalized indefinite strings. By following an approach of Deift and Killip, we establish stability of the absolutely continuous spectra of two model examples of generalized indefinite strings under rather wide perturbations. In particular, one of these results allows us to prove that the absolutely continuous spectrum of the isospectral problem associated with the conservative Camassa–Holm flow in the dispersive regime is essentially supported on the interval $[1/4,\infty )$.

## Introduction

It is well known that the study of spectral types of self-adjoint operators in Hilbert spaces is crucial for understanding the corresponding quantum dynamics. For this reason, even a huge amount of effort has been put in understanding only one of the most basic models — one-body Schrödinger operators with (in a certain sense) decaying potentials. The standard framework to investigate such Schrödinger operators is perturbation theory, where one considers a given operator as an additive (or maybe singular, that is, in the sense of resolvents) perturbation of another operator (for example, the free Hamiltonian) whose spectral properties are rather well understood. According to the Rosenblum–Kato and Birman–Krein theorems, the absolutely continuous spectrum is stable under trace class perturbations. These results are sharp since by the Weyl–von Neumann–Kuroda theorem, absolutely continuous spectrum may be turned into pure point spectrum by a perturbation of any Schatten class weaker than trace class.

On the other hand, by restricting to particular classes of perturbations (for Schrödinger operators, the considered perturbations are usually multiplication operators) one can hope for better results. For example, in their seminal work [14], Deift and Killip proved that the absolutely continuous spectrum of a one-dimensional Schrödinger operator

1.1 $$\begin{aligned} -\frac{d^2}{dx^2} + V(x) \end{aligned}$$

acting in $L^2({{\mathbb {R}}})$ coincides with $[0,\infty )$ as long as *V* is a real-valued potential in $L^2({{\mathbb {R}}})$ (see also [31, 33, 37, 40] for further results and references). A similar statement for multi-dimensional operators is known as *Simon’s conjecture* [42, Conjecture 20.2] and still open, although this area has seen significant progress in the 1990s and 2000s. We are not attempting to give an overview of this topic (which would be a rather difficult task) but only point to the review articles [15] and [41, 42], where further references can be found.

Our aim here is to investigate spectral types, or more precisely, the absolutely continuous part of the spectrum of *generalized indefinite strings*, introduced recently in [20]. We recall briefly (see Sect. 2 for further details) that a generalized indefinite string is a triple $(L,\omega ,\upsilon )$ such that $L\in (0,\infty ]$, $\omega$ is a real distribution in $H^{-1}_{{\mathrm {loc}}}[0,L)$ and $\upsilon$ is a non-negative Borel measure on [0, *L*). Associated with such a triple is the ordinary differential equation of the form

1.2 $$\begin{aligned} -f'' = z\, \omega f + z^2 \upsilon f \end{aligned}$$

on [0, *L*), where *z* is a complex spectral parameter. Spectral problems of this type are of interest for at least two reasons. Firstly, they constitute a canonical model for operators with simple spectrum; see [20, 22]. Secondly, they are of relevance in connection with certain completely integrable nonlinear wave equations (most prominently, the Camassa–Holm equation [9]), for which these kinds of spectral problems arise as isospectral problems.

Results on spectral types, even for the special case of a Krein string

1.3 $$\begin{aligned} -f'' = z\, \omega f, \end{aligned}$$

that is, when $\omega$ is a non-negative Borel measure and $\upsilon$ vanishes identically, are rather scarce (however, let us mention the recent paper by Bessonov and Denisov [5]). The main reason for this lies in the fact that the spectral parameter appears in the *wrong* place, which does not allow to view (1.3) as an additive perturbation immediately. One of the standard approaches here is to transform the differential equation (1.3) into Schrödinger form by means of a Liouville transformation and then apply the well-developed spectral theory for one-dimensional Schrödinger operators. However, this immediately requires strictly positive and sufficiently smooth weights $\omega$, assumptions which are too restrictive for our applications. In this context, let us also mention that spectral theory of *indefinite strings*, that is, when the coefficient $\omega$ in (1.3) is a real-valued Borel measure, is significantly more intricate when compared to the case of Krein strings (see [26, 34] for example).

Our approach to the absolutely continuous spectrum of generalized indefinite strings follows the elegant ideas of Deift and Killip [14]. In order to implement their approach, we need two main ingredients. The first ingredient is a continuity property for the correspondence between generalized indefinite strings and their associated Weyl–Titchmarsh functions (see Sect. 2 for more details) obtained in [20, Proposition 6.2]. The relevance of the Weyl–Titchmarsh function here stems from the fact that a measure $\mu$ in a certain integral representation for this function is a *spectral measure* for (1.2). More precisely, this means that the operator part of a self-adjoint linear relation associated with (1.2) is unitarily equivalent to multiplication with the independent variable in $L^2({{\mathbb {R}}};\mu )$. In particular, the absolutely continuous spectrum of (1.2) can be identified with the topological support of the absolutely continuous part of the measure $\mu$.

The second ingredient is a so-called *trace formula*, which provides a relation between the spectral/scattering data and the coefficients in the differential equation, and hence allows to *control* the spectral measure by means of the coefficients. For Schrödinger operators (1.1), such relations have been discovered by Faddeev and Zakharov [25] (see also [7]) in connection with the Korteweg–de Vries equation, where they give rise to conserved quantities. Generalized indefinite strings on the other side are of the same importance for the conservative Camassa–Holm flow [10, 13, 17–19, 30] as the one-dimensional Schrödinger operator (1.1) for the Korteweg–de Vries equation. In particular, trace formulas for (1.2) are of special interest in this connection because they give rise to conserved quantities of the flow. Under the rather restrictive assumptions that $\upsilon$ vanishes identically and $\omega$ is a sufficiently smooth and positive function, such relations have been established before (see, for example, [12]). Removing the positivity assumption, however, immediately creates an (in general) infinite number of negative eigenvalues for (1.2) and drastically complicates the situation. For this reason, the derivation of corresponding trace formulas for generalized indefinite strings will require more efforts (see Lemma 5.2 and Lemma 6.1).

In regard to the conservative Camassa–Holm flow, our results concern the absolutely continuous spectrum of the corresponding isospectral problem

1.4 $$\begin{aligned} - g'' + \frac{1}{4}g&= z\,\omega \, g + z^2 \upsilon \, g,&\omega&= u - u'', \end{aligned}$$

on the real line. We will show in Sect. 7 that under the assumption that *u* is a real-valued function on ${{\mathbb {R}}}$ such that $u-1$ belongs to $H^1({{\mathbb {R}}})$ and $\upsilon$ is a non-negative finite Borel measure on ${{\mathbb {R}}}$, the essential spectrum and the absolutely continuous spectrum of a self-adjoint realization of (1.4) coincide with the interval $[1/4,\infty )$. In the special case when $\omega -1$ is a measure with strong enough decay and $\upsilon$ vanishes identically, this result has been established before by Bennewitz, Brown and Weikard [3, 4]. Under these additional restrictions, the essential spectrum is in fact purely absolutely continuous, which will not continue to hold for our set of coefficients. Our assumptions on the coefficients cover a natural phase space for the conservative Camassa–Holm flow in the dispersive regime (see [8, 27, 29]), which comprises the Camassa–Holm equation [11] as well as the two-component Camassa–Holm system [10, 13, 30]. Note here that the additional coefficient $\upsilon$ is related to the energy measure $\mu$ from [8, 27, 29] in a simple way, essentially via

1.5 $$\begin{aligned} \mu (B) = \upsilon (B) + \int _B (u(x)-1)^2 + u'(x)^2 dx \end{aligned}$$

for every Borel set $B\subseteq {{\mathbb {R}}}$. The function $\rho$ that appears in the formulation of the two-component Camassa–Holm system is simply the square root of the Radon–Nikodym derivative of the absolutely continuous part of $\upsilon$.

In conclusion, let us sketch the content of the article. Section 2 is of preliminary character and collects necessary notions and facts from the spectral theory of generalized indefinite strings. Section 3 contains the statements of our main results (Theorems 3.1 and 3.2) about the absolutely continuous spectrum for certain classes of generalized indefinite strings, which are rather strong perturbations of explicitly solvable models (Example A and Example B). Although we will not present the necessary details here, these perturbations can indeed be interpreted in a certain way as additive perturbations, which are only of Hilbert–Schmidt class in general however. Our main theorems seem to be new even for the special case of Krein strings and will be proved in Sects. 5 and 6, respectively, after deriving some useful auxiliary facts in Sect. 4. Even though we will generally follow the simple method by Deift and Killip from [14], the necessary ingredients are not as readily available for our spectral problem and need to be established first. In Sect. 7, we will then show how corresponding results for the spectral problem (1.4) can be derived readily from Theorem 3.2. The final section provides another application of our results to one-dimensional Hamiltonians with $\delta '$-interactions.

### Notation

We conclude the introduction by defining several spaces of functions and distributions. For every fixed $$L\in (0,\infty ]$$, we denote with $$H^1_{{\mathrm {loc}}}[0,L)$$, $$H^1[0,L)$$ and $$H^1_{{\mathrm {c}}}[0,L)$$ the usual Sobolev spaces. To be precise, this means

1.6 $$\begin{aligned} H^1_{{\mathrm {loc}}}[0,L)&= \lbrace f\in AC_{{\mathrm {loc}}}[0,L) \,|\, f'\in L^2_{{\mathrm {loc}}}[0,L) \rbrace , \end{aligned}$$

1.7 $$\begin{aligned} H^1[0,L)&= \lbrace f\in H^1_{{\mathrm {loc}}}[0,L) \,|\, f,\, f'\in L^2[0,L) \rbrace , \end{aligned}$$

1.8 $$\begin{aligned} H^1_{{\mathrm {c}}}[0,L)&= \lbrace f\in H^1[0,L) \,|\, \mathrm {supp}(f) \text { compact in } [0,L) \rbrace . \end{aligned}$$

The space of distributions $H^{-1}_{{\mathrm {loc}}}[0,L)$ is the topological dual of $H^1_{{\mathrm {c}}}[0,L)$. One notes that the mapping ${\mathsf {q}}\mapsto \chi$, defined by

1.9 $$\begin{aligned} \chi (h) = - \int _0^L {\mathsf {q}}(x)h'(x)dx, \quad h\in H^1_{{\mathrm {c}}}[0,L), \end{aligned}$$

establishes a one-to-one correspondence between $L^2_{{\mathrm {loc}}}[0,L)$ and $H^{-1}_{{\mathrm {loc}}}[0,L)$. The unique function ${\mathsf {q}}\in L^2_{{\mathrm {loc}}}[0,L)$ corresponding to some distribution $\chi \in H^{-1}_{{\mathrm {loc}}}[0,L)$ in this way will be referred to as *the normalized anti-derivative* of $\chi$. We say that a distribution in $H^{-1}_{{\mathrm {loc}}}[0,L)$ is *real* if its normalized anti-derivative is real-valued almost everywhere on [0, *L*).

A particular kind of distribution in $H^{-1}_{{\mathrm {loc}}}[0,L)$ arises from Borel measures on the interval [0, *L*). In fact, if $\chi$ is a complex-valued Borel measure on [0, *L*), then we will identify it with the distribution in $H^{-1}_{{\mathrm {loc}}}[0,L)$ given by

1.10 $$\begin{aligned} h \mapsto \int _{[0,L)} h\,d\chi . \end{aligned}$$

The normalized anti-derivative ${\mathsf {q}}$ of such a $\chi$ is simply given by the left-continuous distribution function

1.11 $$\begin{aligned} {\mathsf {q}}(x)=\int _{[0,x)}d\chi \end{aligned}$$

for almost all $x\in [0,L)$, as an integration by parts (use, for example, [6, Exercise 5.8.112], [28, Theorem 21.67]) shows.

In order to obtain a self-adjoint realization of the differential equation (1.2) in a suitable Hilbert space later, we also introduce the function space

1.12 $$\begin{aligned} {\dot{H}}^1[0,L)&= {\left\{ \begin{array}{ll} \lbrace f\in H^1_{{\mathrm {loc}}}[0,L) \,|\, f'\in L^2[0,L),~ \lim _{x\rightarrow L} f(x) = 0 \rbrace , &{} L<\infty , \\ \lbrace f\in H^1_{{\mathrm {loc}}}[0,L) \,|\, f'\in L^2[0,L) \rbrace , &{} L=\infty , \end{array}\right. } \end{aligned}$$

as well as the linear subspace

1.13 $$\begin{aligned} {\dot{H}}^1_0[0,L)&= \lbrace f\in {\dot{H}}^1[0,L)\,|\, f(0) = 0 \rbrace , \end{aligned}$$

which turns into a Hilbert space when endowed with the scalar product

1.14 $$\begin{aligned} \langle f , g \rangle _{{\dot{H}}^1_0[0,L)} = \int _0^L f'(x) g'(x)^*dx, \quad f,\, g\in {\dot{H}}^1_0[0,L). \end{aligned}$$

Here and henceforth, we will use a star to denote complex conjugation. The space $${\dot{H}}^1_0[0,L)$$ can be viewed as a completion with respect to the norm induced by (1.14) of the space of all smooth functions which have compact support in (0, *L*). In particular, the space $${\dot{H}}^1_0[0,L)$$ coincides algebraically and topologically with the usual Sobolev space $$H^1_0[0,L)$$ when *L* is finite.

## Generalized Indefinite Strings

A generalized indefinite string is a triple $(L,\omega ,\upsilon )$ such that $L\in (0,\infty ]$, $\omega$ is a real distribution in $H^{-1}_{{\mathrm {loc}}}[0,L)$ and $\upsilon$ is a non-negative Borel measure on the interval [0, *L*). Associated with such a generalized indefinite string is the inhomogeneous differential equation

2.1 $$\begin{aligned} -f'' = z\, \omega f + z^2 \upsilon f + \chi , \end{aligned}$$

where $\chi$ is a distribution in $H^{-1}_{{\mathrm {loc}}}[0,L)$ and *z* is a complex spectral parameter. Of course, this differential equation has to be understood in a weak sense: A solution of (2.1) is a function $f\in H^1_{{\mathrm {loc}}}[0,L)$ such that

2.2 $$\begin{aligned} \Delta _f h(0) + \int _{0}^L f'(x) h'(x) dx = z\, \omega (fh) + z^2 \upsilon (fh) + \chi (h), \quad h\in H^1_{{\mathrm {c}}}[0,L), \end{aligned}$$

for some constant $\Delta _f\in {\mathbb {C}}$. In this case, the constant $\Delta _f$ is uniquely determined and will always be denoted with $f'(0-)$ for apparent reasons. With this notion of solution, common basic existence and uniqueness results for the inhomogeneous differential equation (2.1) are available; see [20, Section 3].

The differential equation (2.1) gives rise to a self-adjoint linear relation in a suitable Hilbert space. In order to introduce this object, we consider the space

2.3 $$\begin{aligned} {\mathcal {H}}= {\dot{H}}^1_0[0,L)\times L^2([0,L);\upsilon ), \end{aligned}$$

which turns into a Hilbert space when endowed with the scalar product

2.4 $$\begin{aligned} \langle f , g \rangle _{{\mathcal {H}}} = \int _0^L f_1'(x) g_1'(x)^*dx + \int _{[0,L)} f_2(x) g_2(x)^*d\upsilon (x), \quad f,\, g\in {\mathcal {H}}. \end{aligned}$$

The respective components of some vector $f\in {\mathcal {H}}$ are hereby always denoted by adding subscripts, that is, with $f_1$ and $f_2$. Now the linear relation $\mathrm {T}$ in the Hilbert space ${\mathcal {H}}$ is defined by saying that some pair $(f,g)\in {\mathcal {H}}\times {\mathcal {H}}$ belongs to $\mathrm {T}$ if and only if the two equations

2.5 $$\begin{aligned} -f_1''&=\omega g_{1} + \upsilon g_{2},&\upsilon f_2&=\upsilon g_{1}, \end{aligned}$$

hold. In order to be precise, the right-hand side of the first equation in (2.5) has to be understood as the $H^{-1}_{{\mathrm {loc}}}[0,L)$ distribution given by

2.6 $$\begin{aligned} h \mapsto \omega (g_1h) + \int _{[0,L)} g_2 h\, d\upsilon . \end{aligned}$$

Moreover, the second equation in (2.5) holds if and only if $f_2$ is equal to $g_1$ almost everywhere on [0, *L*) with respect to the measure $\upsilon$. The maximally defined linear relation $\mathrm {T}$ is self-adjoint in the Hilbert space ${\mathcal {H}}$ according to [20, Theorem 4.1] and indeed closely related to the differential equation (2.1) since for each $z\in {\mathbb {C}}$, a pair $(f,g)\in {\mathcal {H}}\times {\mathcal {H}}$ belongs to $\mathrm {T}-z$ if and only if the two equations

2.7 $$\begin{aligned} -f_1''&= z\, \omega f_1 +z^2 \upsilon f_1 + \omega g_1 + z\,\upsilon g_1 + \upsilon g_2,&\upsilon f_2 = z\,\upsilon f_1 + \upsilon g_1, \end{aligned}$$

hold. This shows that some $f\in {\mathcal {H}}$ belongs to $\mathrm {ker}(\mathrm {T}-z)$ if and only if $f_1$ is a solution of the homogeneous differential equation

2.8 $$\begin{aligned} -f'' = z\, \omega f + z^2 \upsilon f \end{aligned}$$

and $\upsilon f_2 = z\, \upsilon f_1$. In other words, some $z\in {\mathbb {C}}$ is an eigenvalue of $\mathrm {T}$ if and only if there is a non-trivial solution $\phi$ of the homogeneous differential equation (2.8) such that $\phi$ lies in ${\dot{H}}^1_0[0,L)$ and $z\phi$ lies in $L^2([0,L);\upsilon )$.

A central object in the spectral theory for the linear relation $\mathrm {T}$ is the associated *Weyl–Titchmarsh function* *m*. This function can be defined on ${\mathbb {C}}\backslash {{\mathbb {R}}}$ by

2.9 $$\begin{aligned} m(z) = \frac{\psi '(z,0-)}{z\psi (z,0)},\quad z\in {\mathbb {C}}\backslash {{\mathbb {R}}}, \end{aligned}$$

where $\psi (z,\cdot \,)$ is the unique (up to constant multiples) non-trivial solution of the homogeneous differential equation (2.8) which lies in ${\dot{H}}^1[0,L)$ and $L^2([0,L);\upsilon )$, guaranteed to exist by [20, Lemma 4.2]. It has been shown in [20, Lemma 5.1] that the Weyl–Titchmarsh function *m* is a Herglotz–Nevanlinna function, that is, it is analytic, maps the upper complex half-plane ${\mathbb {C}}_+$ into the closure of the upper complex half-plane and satisfies the symmetry relation

2.10 $$\begin{aligned} m(z)^*= m(z^*), \quad z\in {\mathbb {C}}\backslash {{\mathbb {R}}}. \end{aligned}$$

For this reason, the Weyl–Titchmarsh function *m* admits an integral representation, which takes the form

2.11 $$\begin{aligned} m(z) = c_1 z + c_2 - \frac{1}{Lz} + \int _{{\mathbb {R}}}\frac{1}{\lambda -z} - \frac{\lambda }{1+\lambda ^2}\, d\mu (\lambda ), \quad z\in {\mathbb {C}}\backslash {{\mathbb {R}}}, \end{aligned}$$

for some constants $c_1$, $c_2\in {{\mathbb {R}}}$ with $c_1\ge 0$ and a non-negative Borel measure $\mu$ on ${{\mathbb {R}}}$ with $\mu (\lbrace 0\rbrace )=0$ for which the integral

2.12 $$\begin{aligned} \int _{{\mathbb {R}}}\frac{d\mu (\lambda )}{1+\lambda ^2} \end{aligned}$$

is finite. Here we employ the convention that whenever an *L* appears in a denominator, the corresponding fraction has to be interpreted as zero if *L* is infinite.

The measure $\mu$ turns out to be a *spectral measure* for the linear relation $\mathrm {T}$ in the sense that the operator part of $\mathrm {T}$ is unitarily equivalent to multiplication with the independent variable in $L^2({{\mathbb {R}}};\mu )$; see [20, Theorem 5.8]. Of course, this establishes an immediate connection between the spectral properties of the linear relation $\mathrm {T}$ and the measure $\mu$. For example, the spectrum of $\mathrm {T}$ coincides with the topological support of $\mu$ and thus can be read off the singularities of *m* (more precisely, the function *m* admits an analytic continuation away from the spectrum of $\mathrm {T}$).

For the sake of simplicity, we shall always mean the spectrum of the corresponding linear relation when we speak of the spectrum of a generalized indefinite string in the following. The same convention applies to the various spectral types.

## Absolutely Continuous Spectrum

In general, any kind of (simple) spectrum can arise from a generalized indefinite string; see [20, Theorem 6.1]. Here, we are interested in the absolutely continuous spectrum of a particular class of generalized indefinite strings, which are suitable perturbations of the following explicitly solvable case.

### Example A

Let $S_0$ be the generalized indefinite string $(L_0,\omega _0,\upsilon _0)$ such that $L_0$ is infinite, the distribution $\omega _0$ is given via its normalized anti-derivative ${\mathsf {w}}_0$ by

3.1 $$\begin{aligned} {\mathsf {w}}_0(x) = x, \quad x\in [0,\infty ), \end{aligned}$$

and the measure $\upsilon _0$ vanishes identically. We note that under these assumptions, the corresponding differential equation (2.8) simply reduces to

3.2 $$\begin{aligned} - f'' = zf. \end{aligned}$$

For every $z\in {\mathbb {C}}\backslash [0,\infty )$, the function $\psi _0(z,\cdot \,)$ given by^1^

3.3 $$\begin{aligned} \psi _0(z,x) = \mathrm {e}^{\mathrm {i}\sqrt{z}x}, \quad x\in [0,\infty ), \end{aligned}$$

is a solution of this differential equation which lies in ${\dot{H}}^1[0,\infty )$. Consequently, the corresponding Weyl–Titchmarsh function $m_0$ is given explicitly by

3.4 $$\begin{aligned} m_0(z) = \frac{\psi _0'(z,0-)}{z\psi _0(z,0)} = \frac{\mathrm {i}}{\sqrt{z}}, \quad z\in {\mathbb {C}}\backslash {{\mathbb {R}}}. \end{aligned}$$

This guarantees that the spectrum of $S_0$ is purely absolutely continuous and coincides with the interval $[0,\infty )$.

In particular, the essential spectrum of $S_0$ coincides with the interval $[0,\infty )$ and the absolutely continuous spectrum of $S_0$ is essentially supported on $[0,\infty )$. The latter means that every subset of $[0,\infty )$ with positive Lebesgue measure has positive measure with respect to the corresponding spectral measure $\mu _0$. It turns out that these two properties continue to hold under a rather wide class of perturbations.

### Theorem 3.1

Let *S* be a generalized indefinite string $(L,\omega ,\upsilon )$ such that *L* is infinite and

3.5 $$\begin{aligned} \int _0^\infty \left| {\mathsf {w}}(x) - c - \eta x\right| ^2 dx + \int _{[0,\infty )} d\upsilon&< \infty \end{aligned}$$

for a real constant *c* and a positive constant $\eta$, where ${\mathsf {w}}$ is the normalized anti-derivative of $\omega$. Then the essential spectrum of *S* coincides with the interval $[0,\infty )$ and the absolutely continuous spectrum of *S* is essentially supported on $[0,\infty )$.

A proof for this result will be given in Sect. 5. In view of the applications we have in mind (see Sect. 7), we are furthermore interested in perturbations of another explicitly solvable case involving a positive parameter $\alpha$.

### Example B

Let $S_\alpha$ be the generalized indefinite string $(L_\alpha ,\omega _\alpha ,\upsilon _\alpha )$ such that $L_\alpha$ is infinite, the distribution $\omega _\alpha$ is given via its normalized anti-derivative ${\mathsf {w}}_\alpha$ by

3.6 $$\begin{aligned} {\mathsf {w}}_\alpha (x) = \frac{x}{1+2\sqrt{\alpha }x}, \quad x\in [0,\infty ), \end{aligned}$$

and the measure $\upsilon _\alpha$ vanishes identically. We note that under these assumptions, the corresponding differential equation (2.8) simply reduces to

3.7 $$\begin{aligned} - f''(x) = \frac{z}{(1+2\sqrt{\alpha }x)^2} f(x),\quad x\in [0,\infty ). \end{aligned}$$

For every $z\in {\mathbb {C}}\backslash [\alpha ,\infty )$, the function $\psi _\alpha (z,\cdot \,)$ given by

3.8 $$\begin{aligned} \psi _\alpha (z,x) = (1+2\sqrt{\alpha }x)^{\mathrm {i}\frac{\sqrt{z-\alpha }}{2\sqrt{\alpha }} + \frac{1}{2}}, \quad x\in [0,\infty ), \end{aligned}$$

is a solution of this differential equation which lies in ${\dot{H}}^1[0,\infty )$. Consequently, the corresponding Weyl–Titchmarsh function $m_\alpha$ is given explicitly by

3.9 $$\begin{aligned} m_\alpha (z) = \frac{\psi _\alpha '(z,0-)}{z\psi _\alpha (z,0)} = \frac{\mathrm {i}}{\sqrt{z-\alpha }+\mathrm {i}\sqrt{\alpha }}, \quad z\in {\mathbb {C}}\backslash {{\mathbb {R}}}. \end{aligned}$$

This guarantees that the spectrum of $S_\alpha$ is purely absolutely continuous and coincides with the interval $[\alpha ,\infty )$.

In particular, the essential spectrum of $S_\alpha$ coincides with the interval $[\alpha ,\infty )$ and the absolutely continuous spectrum of $S_\alpha$ is essentially supported on $[\alpha ,\infty )$. These two properties are again preserved under a rather wide class of perturbations.

### Theorem 3.2

Let *S* be a generalized indefinite string $(L,\omega ,\upsilon )$ such that *L* is infinite and

3.10 $$\begin{aligned} \int _0^\infty \Bigl | {\mathsf {w}}(x) - c - \frac{\eta x}{1+2\sqrt{\alpha }x}\Bigr |^2 x\, dx + \int _{[0,\infty )} x\, d\upsilon (x)&< \infty \end{aligned}$$

for a real constant *c* and positive constants $\alpha$ and $\eta$, where ${\mathsf {w}}$ is the normalized anti-derivative of $\omega$. Then the essential spectrum of *S* coincides with the interval $[\alpha /\eta ,\infty )$ and the absolutely continuous spectrum of *S* is essentially supported on $[\alpha /\eta ,\infty )$.

Although the proof of this result is quite similar to the one for Theorem 3.1 in principle, it will be carried out separately in Sect. 6 due to differences in details.

### Remark 3.3

When the constant $\eta$ in the assumptions of Theorem 3.1 and Theorem 3.2 is negative, then the resulting spectral picture is simply reflected across the imaginary axis. In this case, the essential spectrum of *S* coincides with the interval $(-\infty ,0]$ and $(-\infty ,\alpha /\eta ]$, respectively, and the absolutely continuous spectrum of *S* is essentially supported on $(-\infty ,0]$ and $(-\infty ,\alpha /\eta ]$, respectively.

As a conclusion to this section, let us mention that although we restricted to generalized indefinite strings on infinite intervals here, one can also consider perturbations of similar explicitly solvable cases on finite intervals.

## Auxiliary Facts About Fundamental Systems

Let the triple $(L,\omega ,\upsilon )$ be an arbitrary generalized indefinite string and denote with ${\mathsf {w}}$ the normalized anti-derivative of $\omega$. For every $z\in {\mathbb {C}}$, we introduce the fundamental system of solutions $\theta (z,\cdot \,)$, $\phi (z,\cdot \,)$ of the differential equation (2.8) satisfying the initial conditions

4.1 $$\begin{aligned} \theta (z,0)&= \phi '(z,0-)=1,&\theta '(z,0-)&= \phi (z,0) =0. \end{aligned}$$

As the derivatives of these functions are only locally square integrable in general, we introduce the left-continuous *quasi-derivatives* $\theta ^{[1]}(z,\cdot \,)$, $\phi ^{[1]}(z,\cdot \,)$ on [0, *L*) by

4.2 $$\begin{aligned} \theta ^{[1]}(z,x)&= \theta '(z,0-)+ z\int _0^x {\mathsf {w}}(t)\theta '(z,t)dt - z^2 \int _{[0,x)} \theta (z,t)d\upsilon (t), \end{aligned}$$

4.3 $$\begin{aligned} \phi ^{[1]}(z,x)&= \phi '(z,0-)+ z\int _0^x {\mathsf {w}}(t)\phi '(z,t)dt - z^2 \int _{[0,x)} \phi (z,t)d\upsilon (t), \end{aligned}$$

for $x\in [0,L)$, such that

4.4 $$\begin{aligned} \theta ^{[1]}(z,x)&= \theta '(z,x) + z{\mathsf {w}}(x)\theta (z,x),&\phi ^{[1]}(z,x)&= \phi '(z,x) + z{\mathsf {w}}(x)\phi (z,x), \end{aligned}$$

for almost all $x\in [0,L)$; see [20, Equation (4.12)]. It follows from [20, Corollary 3.5] that the functions

4.5 $$\begin{aligned} z&\mapsto \theta (z,x),&z&\mapsto \theta ^{[1]}(z,x),&z&\mapsto \phi (z,x),&z&\mapsto \phi ^{[1]}(z,x), \end{aligned}$$

are real entire for every fixed $x\in [0,L)$. At the origin, when *z* is zero, one readily infers that our fundamental system is given explicitly by

4.6 $$\begin{aligned} \theta (0,x)&= 1,&\theta ^{[1]}(0,x)&= 0,&\phi (0,x)&= x,&\phi ^{[1]}(0,x)&= 1, \end{aligned}$$

for all $x\in [0,L)$. We now seek to determine the derivatives of these functions with respect to the spectral parameter at the origin. In order to state the following result, let us note that differentiation with respect to the spectral parameter will be denoted with a dot and is always meant to be done after taking quasi-derivatives.

### Proposition 4.1

For every $x\in [0,L)$, we have

4.7 $$\begin{aligned} {\dot{\theta }}(0,x)&= - \int _0^x {\mathsf {w}}(t)dt,&{\dot{\theta }}^{[1]}(0,x)&= 0, \end{aligned}$$

4.8 $$\begin{aligned} {\dot{\phi }}(0,x)&= \int _0^x \int _0^t {\mathsf {w}}(s)ds\, dt - \int _0^x {\mathsf {w}}(t) t\, dt,&{\dot{\phi }}^{[1]}(0,x)&= \int _0^x {\mathsf {w}}(t)dt, \end{aligned}$$

as well as

4.9 $$\begin{aligned} \ddot{\theta }(0,x)&= \biggl (\int _0^x {\mathsf {w}}(t)dt\biggr )^2 - 2 \int _0^x \int _0^t {\mathsf {w}}(s)^2 ds\,dt - 2 \int _0^x \int _{[0,t)} d\upsilon \,dt, \end{aligned}$$

4.10 $$\begin{aligned} \ddot{\theta }^{[1]}(0,x)&= -2 \int _0^x {\mathsf {w}}(t)^2 dt -2 \int _{[0,x)} d\upsilon , \end{aligned}$$

4.11 $$\begin{aligned} \ddot{\phi }^{[1]}(0,x)&= \biggl (\int _0^x {\mathsf {w}}(t)dt\biggr )^2 - 2 \int _0^x {\mathsf {w}}(t)^2 t\, dt -2 \int _{[0,x)} t\, d\upsilon (t). \end{aligned}$$

### Proof

We first note that the second equality in (4.7) follows from (4.2) since the first integral there is zero when *z* is equal to zero. Now consider the matrix function

$$\begin{aligned} Y(z,x) = \begin{pmatrix} \theta (z,x) &{}\quad -z\phi (z,x) \\ -z^{-1}\theta ^{[1]}(z,x) &{} \quad \phi ^{[1]}(z,x) \end{pmatrix}, \quad z\in {\mathbb {C}},~x\in [0,L), \end{aligned}$$

which is well-defined since the function $\theta ^{[1]}(\,\cdot \,,x)$ has a root at zero. It is an immediate consequence of (4.2), (4.3) and (4.4) that *Y* satisfies the integral equation

$$\begin{aligned} Y(z,x) = \begin{pmatrix} 1 &{} 0 \\ 0 &{} 1 \end{pmatrix}&+ z \int _0^x \begin{pmatrix} - {\mathsf {w}}(t) &{} -1 \\ {\mathsf {w}}(t)^2 &{} {\mathsf {w}}(t) \end{pmatrix} Y(z,t) dt \\&+ z \int _{[0,x)} \begin{pmatrix} 0 &{}\quad 0 \\ 1 &{}\quad 0 \end{pmatrix}Y(z,t) d\upsilon (t), \quad x\in [0,L),~z\in {\mathbb {C}}. \end{aligned}$$

For fixed $x\in [0,L)$, differentiating with respect to *z* gives

4.12 $$\begin{aligned} \begin{aligned} {\dot{Y}}(z,x)&= \int _0^x \begin{pmatrix} - {\mathsf {w}}(t) &{} \quad -1 \\ {\mathsf {w}}(t)^2 &{}\quad {\mathsf {w}}(t) \end{pmatrix} Y(z,t) dt + z \int _0^x \begin{pmatrix} - {\mathsf {w}}(t) &{} -1 \\ {\mathsf {w}}(t)^2 &{} \quad {\mathsf {w}}(t) \end{pmatrix} {\dot{Y}}(z,t) dt \\&\qquad + \int _{[0,x)} \begin{pmatrix} 0 &{}\quad 0 \\ 1 &{}\quad 0 \end{pmatrix}Y(z,t) d\upsilon (t) + z \int _{[0,x)} \begin{pmatrix} 0 &{}\quad 0 \\ 1 &{}\quad 0 \end{pmatrix} {\dot{Y}}(z,t) d\upsilon (t), \quad z\in {\mathbb {C}}. \end{aligned}\end{aligned}$$

Evaluating at zero, we end up with

$$\begin{aligned} {\dot{Y}}(0,x) = \int _0^x \begin{pmatrix} - {\mathsf {w}}(t) &{}\quad -1 \\ {\mathsf {w}}(t)^2 &{}\quad {\mathsf {w}}(t) \end{pmatrix} dt + \int _{[0,x)} \begin{pmatrix} 0 &{}\quad 0 \\ 1 &{}\quad 0 \end{pmatrix} d\upsilon (t), \end{aligned}$$

which yields the first equality in (4.7), the second equality in (4.8) as well as (4.10). Differentiating (4.12) once more and evaluating at zero, we obtain

$$\begin{aligned} {\ddot{Y}}(0,x) = 2 \int _0^x \begin{pmatrix} - {\mathsf {w}}(t) &{}\quad -1 \\ {\mathsf {w}}(t)^2 &{}\quad {\mathsf {w}}(t) \end{pmatrix} {\dot{Y}}(0,t) dt + 2 \int _{[0,x)} \begin{pmatrix} 0 &{}\quad 0 \\ 1 &{}\quad 0 \end{pmatrix} {\dot{Y}}(0,t) d\upsilon (t), \end{aligned}$$

which yields the first equality in (4.8), (4.9) as well as (4.11) after performing some integrations by parts. $\square$

Although one could also compute the second derivative of $\phi (\,\cdot \,,x)$ at zero, we omitted to include it because the expression is somewhat lengthy and will not be needed in what follows. In fact, to this end one just needs to note that

4.13 $$\begin{aligned} \ddot{\phi }(z,x) = \int _0^x \ddot{\phi }^{[1]}(z,t) dt - 2 \int _0^x {\mathsf {w}}(t) {\dot{\phi }}(z,t) dt - z \int _0^x {\mathsf {w}}(t) \ddot{\phi }(z,t)dt, \quad z\in {\mathbb {C}}, \end{aligned}$$

and plug in the expressions from Proposition 4.1 upon evaluating at zero.

## Proof of Theorem 3.1

To begin with, let us consider a particular class of generalized indefinite strings. We assume that $(L,\omega ,\upsilon )$ is a generalized indefinite string such that *L* is infinite and there is an $R>0$ such that the normalized anti-derivative ${\mathsf {w}}$ of $\omega$ satisfies

5.1 $$\begin{aligned} {\mathsf {w}}(x) = x \end{aligned}$$

for almost all *x* in $[R,\infty )$ and the measure $\upsilon$ vanishes on $[R,\infty )$. In addition, let us also suppose that ${\mathsf {w}}$ is equal to a piecewise constant function almost everywhere on the interval [0, *R*] and that the support of the measure $\upsilon$ is a finite set. The set of generalized indefinite strings with these properties will be denoted by ${\mathcal {F}}_0$. Under these assumptions, for every *k* in the upper complex half-plane ${\mathbb {C}}_+$, there is a *Jost solution* $f(k,\cdot \,)$ of the differential equation (2.8) with $z=k^2 \in {\mathbb {C}}\backslash [0,\infty )$ such that

5.2 $$\begin{aligned} f(k,x) = \mathrm {e}^{\mathrm {i}k x}, \quad x\in [R,\infty ). \end{aligned}$$

We note that since the function $f(k,\cdot \,)$ clearly lies in ${\dot{H}}^1[0,\infty )$ and $L^2([0,\infty );\upsilon )$, the corresponding Weyl–Titchmarsh function *m* is given by

5.3 $$\begin{aligned} m(k^2) = \frac{f'(k,0-)}{k^2f(k,0)} \end{aligned}$$

as long as $k^2\in {\mathbb {C}}\backslash {{\mathbb {R}}}$. Furthermore, we define the function *a* on ${\mathbb {C}}_+$ via

5.4 $$\begin{aligned} a(k) = \frac{\mathrm {i}k f(k,0)+f'(k,0-)}{2\mathrm {i}k}, \quad k\in {\mathbb {C}}_+, \end{aligned}$$

which can be viewed as the reciprocal transmission coefficient when the differential equation is suitably extended to the full line.

### Lemma 5.1

If the generalized indefinite string $(L,\omega ,\upsilon )$ belongs to ${\mathcal {F}}_0$, then the function *a* has a unique continuation (denoted with *a* as well for simplicity) to an entire function.

### Proof

If $\theta$, $\phi$ denotes the fundamental system of solutions of the differential equation (2.8) as in Sect. 4, then we may write

$$\begin{aligned} f(k,x) = f(k,0)\theta (k^2,x) + f'(k,0-)\phi (k^2,x), \quad x\in [0,\infty ),~k\in {\mathbb {C}}_+. \end{aligned}$$

Upon evaluating this function and its derivative at the point *R*, we get

$$\begin{aligned} \mathrm {e}^{\mathrm {i}k R}&= f(k,0)\theta (k^2,R) + f'(k,0-)\phi (k^2,R), \\ \mathrm {i}k \mathrm {e}^{\mathrm {i}k R} + k^2 R \mathrm {e}^{\mathrm {i}k R}&= f(k,0)\theta ^{[1]}(k^2,R) + f'(k,0-)\phi ^{[1]}(k^2,R), \end{aligned}$$

which we can solve for *f*(*k*, 0) and $f'(k,0-)$ to obtain

5.5 $$\begin{aligned} f(k,0)&= \mathrm {e}^{\mathrm {i}k R}\phi ^{[1]}(k^2,R) - (\mathrm {i}k + k^2 R)\mathrm {e}^{\mathrm {i}k R} \phi (k^2,R), \end{aligned}$$

5.6 $$\begin{aligned} f'(k,0-)&= -\mathrm {e}^{\mathrm {i}k R}\theta ^{[1]}(k^2,R) + (\mathrm {i}k + k^2 R)\mathrm {e}^{\mathrm {i}k R}\theta (k^2,R). \end{aligned}$$

Plugging this into the definition of *a* shows that

5.7 $$\begin{aligned} \begin{aligned} \mathrm {e}^{-\mathrm {i}k R} a(k)&=\frac{1-\mathrm {i}k R}{2} \theta (k^2,R) - \frac{1}{2\mathrm {i}k} \theta ^{[1]}(k^2,R) \\&\qquad -\frac{\mathrm {i}k+ k^2 R}{2} \phi (k^2,R) +\frac{1}{2} \phi ^{[1]}(k^2,R), \quad k\in {\mathbb {C}}_+. \end{aligned} \end{aligned}$$

In view of the second equality in (4.6), this identity guarantees that the function *a* has a unique continuation to an entire function. $\square$

Even more, the right-hand side of (5.7) is actually a polynomial in *k* due to our assumptions on the supports of $\omega$ and $\upsilon$ on [0, *R*]. This entails that the function *a* has only a finite number of zeros, none of which lie on the real axis. In fact, in order to verify this, we first define the function *b* on ${\mathbb {C}}_+$ via

5.8 $$\begin{aligned} b(k) = \frac{\mathrm {i}k f(k,0) - f'(k,0-)}{2\mathrm {i}k}, \quad k\in {\mathbb {C}}_+. \end{aligned}$$

Similarly as before, we see that *b* can be continued to an entire function because of the identity

5.9 $$\begin{aligned} \begin{aligned} \mathrm {e}^{-\mathrm {i}k R} b(k)&= - \frac{1 - \mathrm {i}k R}{2} \theta (k^2,R) + \frac{1}{2\mathrm {i}k} \theta ^{[1]}(k^2,R) \\&\quad \qquad - \frac{\mathrm {i}k+ k^2 R}{2} \phi (k^2,R) + \frac{1}{2} \phi ^{[1]}(k^2,R), \quad k\in {\mathbb {C}}_+. \end{aligned}\end{aligned}$$

Now it is a straightforward computation to verify that for real *k*, we have

5.10 $$\begin{aligned} a(k)^*&= a(-k),&b(k)^*&= b(-k), \end{aligned}$$

as well as

5.11 $$\begin{aligned} |a(k)|^2 = |b(k)|^2 + 1, \end{aligned}$$

which guarantees that *a* has no zeros on the real axis. Furthermore, all zeros in the upper complex half-plane ${\mathbb {C}}_+$ necessarily have to lie on the imaginary axis. In fact, if *k* was a zero of *a* in ${\mathbb {C}}_+$ that does not lie on the imaginary axis, then $k^2\in {\mathbb {C}}\backslash {{\mathbb {R}}}$ and (5.4) would imply

5.12 $$\begin{aligned} f'(k,0-) = -\mathrm {i}k f(k,0). \end{aligned}$$

By using (5.3), this would allow us to compute the imaginary part

5.13 $$\begin{aligned} \mathrm {Im}\, m(k^2) = \mathrm {Im}\, \frac{f'(k,0-)}{k^2f(k,0)} = \mathrm {Im}\, \frac{1}{\mathrm {i}k} = -\frac{\mathrm {Re}\, k}{|k|^2} = - \frac{\mathrm {Im}\, k^2}{2|k|^2\mathrm {Im}\,k}, \end{aligned}$$

which is a contradiction to the fact that *m* is a Herglotz–Nevanlinna function. As this proves that all zeros in ${\mathbb {C}}_+$ indeed lie on the imaginary axis, we may enumerate them, repeated according to multiplicity (it can be shown that they are simple but we do not need this here), by $\mathrm {i}\kappa _1,\ldots ,\mathrm {i}\kappa _N$ for some positive constants $\kappa _1,\ldots ,\kappa _N$. With this notation, let us state the following result.

### Lemma 5.2

If the generalized indefinite string $(L,\omega ,\upsilon )$ belongs to ${\mathcal {F}}_0$, then we have the identity

5.14 $$\begin{aligned} \frac{4}{3} \sum _{n=1}^N \frac{1}{\kappa _n^3} + \frac{2}{\pi } \int _{{{\mathbb {R}}}} \frac{1}{k^4} \log |a(k)| dk =\int _0^\infty |{\mathsf {w}}(x) - x |^2 dx + \int _{[0,\infty )} d\upsilon . \end{aligned}$$

### Proof

From the representation (5.7) for *a*, together with the formulas in Proposition 4.1 for the fundamental system $\theta$, $\phi$, we see that

$$\begin{aligned} a(0)&= 1,&a'(0)&= 0,&a''(0)&= 0, \end{aligned}$$

and after a cumbersome but straightforward computation furthermore that

5.15 $$\begin{aligned} a'''(0) = -3\mathrm {i}\biggl (\int _0^\infty |{\mathsf {w}}(x)-x|^2 dx + \int _{[0,\infty )} d\upsilon \biggr ). \end{aligned}$$

In particular, this yields the Taylor expansion

$$\begin{aligned} a(k) = 1 - k^3 \frac{\mathrm {i}}{2} \biggl (\int _0^\infty |{\mathsf {w}}(x)-x|^2 dx + \int _{[0,\infty )} d\upsilon \biggr ) + {\mathcal {O}}(k^4), \qquad k\rightarrow 0, \end{aligned}$$

around zero, which entails that

5.16 $$\begin{aligned} \log |a(k)| = {\mathcal {O}}(k^4) \end{aligned}$$

as $k\rightarrow 0$ on the real line.

Since the function *a* is of bounded type in the upper complex half-plane, it admits a Nevanlinna factorization [38, Theorem 6.13] of the form

$$\begin{aligned} a(k) = C \prod _{n=1}^N \frac{\mathrm {i}\kappa _n - k}{\mathrm {i}\kappa _n + k}\exp \biggl \{-\mathrm {i}\beta k + \frac{1}{\pi \mathrm {i}} \int _{{\mathbb {R}}}\biggl (\frac{1}{t-k}-\frac{t}{1+t^2}\biggr )\log |a(t)|dt\biggr \}, \quad k\in {\mathbb {C}}_+, \end{aligned}$$

for some real constant $\beta \in {{\mathbb {R}}}$ (in fact, it is not difficult to show that $\beta = - R$) and a complex constant $C\in {\mathbb {C}}$ with modulus one. Upon differentiating this, we obtain

$$\begin{aligned} \frac{a'(k)}{a(k)} = -\mathrm {i}\beta + \frac{1}{\pi \mathrm {i}} \int _{{\mathbb {R}}}\frac{1}{(t-k)^2} \log |a(t)|dt + \sum _{n=1}^N \frac{2\mathrm {i}\kappa _n}{\kappa _n^2+k^2} \end{aligned}$$

for all $k\in {\mathbb {C}}_+$ close enough to zero (so that *a*(*k*) is non-zero). After differentiating two more times, we get

$$\begin{aligned}&\frac{a'''(k)}{a(k)} - 3\frac{a'(k)a''(k)}{a(k)^2} + 2\frac{a'(k)^3}{a(k)^3} \\&\qquad \qquad = \frac{6}{\pi \mathrm {i}} \int _{{\mathbb {R}}}\frac{1}{(t-k)^4} \log |a(t)|dt - {4}{\mathrm {i}}\sum _{n=1}^N \frac{\kappa _n(\kappa _n^2 - k^2)^2 - 4\kappa _n k^4}{(\kappa _n^2+k^2)^4}, \end{aligned}$$

again, as long as $k\in {\mathbb {C}}_+$ is close enough to zero. Now we obtain identity (5.14) upon letting *k* tend to zero, employing (5.15) and noting that the limit of the integral on the right-hand side exists because of the asymptotics (5.16). $\square$

Note that both terms on the left-hand side of the identity (5.14) are non-negative in view of (5.11). In particular, this observation will allow us to obtain an estimate on the negative eigenvalues of the corresponding self-adjoint realization. To this end, we first point out that there are only finitely many such eigenvalues. More precisely, we see from (5.5) and (5.6) that the right-hand side of (5.3) is a rational function. This implies that the Weyl–Titchmarsh function *m* has a continuation to a meromorphic function on ${\mathbb {C}}\backslash [0,\infty )$ with only finitely many poles. As a consequence, the negative spectrum of $(L,\omega ,\upsilon )$ consists only of finitely many eigenvalues. Upon enumerating these eigenvalues by $\lambda _1,\ldots ,\lambda _{K}$ with increasing modulus, we obtain the following Lieb–Thirring-type bound.

### Corollary 5.3

If the generalized indefinite string $(L,\omega ,\upsilon )$ belongs to ${\mathcal {F}}_0$, then we have the estimate

5.17 $$\begin{aligned} \frac{4}{3} \sum _{i=1}^{K} \frac{1}{|\lambda _i|^{3/2}} \le \int _0^\infty |{\mathsf {w}}(x) - x |^2 dx + \int _{[0,\infty )} d\upsilon . \end{aligned}$$

### Proof

We may assume that there are negative eigenvalues. Since the function *m* is a Herglotz–Nevanlinna function, we see from (5.3) that the function

$$\begin{aligned} \kappa \mapsto -\frac{f'(\mathrm {i}\kappa ,0-)}{\kappa ^2 f(\mathrm {i}\kappa ,0)} \end{aligned}$$

is real-valued, continuous and strictly decreasing for positive $\kappa$ away from the poles $\sqrt{-\lambda _1},\ldots ,\sqrt{-\lambda _K}$. Because of this, we can find a positive $\kappa <\sqrt{-\lambda _1}$ such that

5.18 $$\begin{aligned} -\frac{f'(\mathrm {i}\kappa ,0-)}{\kappa ^2 f(\mathrm {i}\kappa ,0)} = -\frac{1}{\kappa }. \end{aligned}$$

Since this means that $\mathrm {i}\kappa$ is a zero of *a*, there is an index $n(1)\in \lbrace 1,\ldots ,N\rbrace$ such that $\kappa =\kappa _{n(1)}$ and thus $\kappa _{n(1)}<\sqrt{-\lambda _{1}}$. If $K>1$ and $\lambda _{i-1}$, $\lambda _{i}$ are two consecutive eigenvalues for some $i\in \{2,\ldots ,K\}$, then we can find a positive $\kappa$ between $\sqrt{-\lambda _{i-1}}$ and $\sqrt{-\lambda _{i}}$ such that (5.18) holds true. As before, we see that $\mathrm {i}\kappa$ is a zero of *a* so that there is an index $n(i)\in \lbrace 1,\ldots ,N\rbrace$ such that $\kappa =\kappa _{n(i)}$ and thus also $\kappa _{n(i)}<\sqrt{-\lambda _{i}}$. In conclusion, this shows that

$$\begin{aligned} \frac{4}{3} \sum _{i=1}^{K} \frac{1}{|\lambda _i|^{3/2}} \le \frac{4}{3} \sum _{i=1}^{K} \frac{1}{\kappa _{n(i)}^3} \le \frac{4}{3} \sum _{n=1}^N \frac{1}{\kappa _n^3}, \end{aligned}$$

which yields the claim upon invoking Lemma 5.2. $\square$

The next ingredient for our proof will be an estimate on the absolutely continuous spectrum of $(L,\omega ,\upsilon )$. To this end, we first note that we have

5.19 $$\begin{aligned} \mathrm {i}k\, m(k^2) = \frac{b(k)-a(k)}{b(k) + a(k)} \end{aligned}$$

for all $k\in {\mathbb {C}}_+$ with $k^2\in {\mathbb {C}}\backslash {{\mathbb {R}}}$. Since the functions *a* and *b* are entire and satisfy the properties (5.10) and (5.11) on the real line, one can conclude that the spectrum of $(L,\omega ,\upsilon )$ on the interval $[0,\infty )$ is purely absolutely continuous with the corresponding spectral measure $\mu$ given by

5.20 $$\begin{aligned} \mu (B) = \int _B \varrho (\lambda ) d\lambda \end{aligned}$$

for every Borel set $B\subseteq [0,\infty )$, where $\varrho$ is defined by

5.21 $$\begin{aligned} \varrho (\lambda ) = \lim _{\varepsilon \rightarrow 0} \frac{1}{\pi }\mathrm {Im}\, m(\lambda +\mathrm {i}\varepsilon ) = \frac{1}{\pi \sqrt{\lambda }|b(\sqrt{\lambda }) + a(\sqrt{\lambda })|^2}, \quad \lambda \in (0,\infty ). \end{aligned}$$

We note that the function $\varrho$ is continuous and positive on $(0,\infty )$.

### Corollary 5.4

If the generalized indefinite string $(L,\omega ,\upsilon )$ belongs to ${\mathcal {F}}_0$, then for every compact subset $\Omega$ of $(0,\infty )$ we have the estimate

5.22 $$\begin{aligned} - \frac{1}{\pi }\int _\Omega \log \biggl (\varrho (\lambda ) \frac{C_\Omega \lambda ^3}{\sqrt{\lambda }}\biggl ) \frac{\sqrt{\lambda }}{\lambda ^3} d\lambda \le \int _0^\infty |{\mathsf {w}}(x) - x |^2 dx + \int _{[0,\infty )} d\upsilon , \end{aligned}$$

where $C_\Omega = 4\pi (\min \,\Omega )^{-2}$ is a positive constant.

### Proof

For every positive *k*, we first compute that

$$\begin{aligned} \left| 1- \frac{b(k)-a(k)}{b(k)+a(k)}\right| ^2 = \frac{4 |a(k)|^2}{|b(k)+a(k)|^2} = 4\pi k |a(k)|^2 \varrho (k^2) \end{aligned}$$

and on the other side that

$$\begin{aligned} \left| 1- \frac{b(k)-a(k)}{b(k)+a(k)}\right| \ge \mathrm {Re}\left( 1- \frac{b(k)-a(k)}{b(k)+a(k)}\right) = 1+ \frac{1}{|b(k)+a(k)|^2} \ge 1. \end{aligned}$$

In combination, this gives the bound

$$\begin{aligned} \frac{1}{|a(\sqrt{\lambda })|^2} \le 4\pi \sqrt{\lambda } \varrho (\lambda ) \le C_\Omega \lambda ^{5/2} \varrho (\lambda ) \end{aligned}$$

as long as $\lambda \in \Omega$, which allows us to estimate the integral

$$\begin{aligned} - \frac{1}{\pi } \int _\Omega \log \biggl (\varrho (\lambda ) \frac{C_\Omega \lambda ^3}{\sqrt{\lambda }}\biggl ) \frac{\sqrt{\lambda }}{\lambda ^3} d\lambda \le \frac{1}{\pi } \int _\Omega \log |a(\sqrt{\lambda })|^2 \frac{\sqrt{\lambda }}{\lambda ^3}d\lambda . \end{aligned}$$

Upon employing a substitution, we can further bound this by

$$\begin{aligned} \frac{2}{\pi } \int _{\sqrt{\min \Omega }}^{\sqrt{\max \Omega }} \log |a(k)|^2 \frac{1}{k^4} dk \le \frac{4}{\pi } \int _{0}^{\infty } \log |a(k)| \frac{1}{k^4} dk = \frac{2}{\pi } \int _{{{\mathbb {R}}}} \log |a(k)| \frac{1}{k^4} dk, \end{aligned}$$

which yields the claim in view of Lemma 5.2. $\square$

With these auxiliary facts, we are now in position to prove our first theorem.

### Proof of Theorem 3.1

Let us assume for now that *S* is a generalized indefinite string $(L,\omega ,\upsilon )$ such that *L* is infinite and

$$\begin{aligned} \int _0^\infty \left| {\mathsf {w}}(x) - x\right| ^2 dx + \int _{[0,\infty )} d\upsilon&< \infty , \end{aligned}$$

where ${\mathsf {w}}$ is the normalized anti-derivative of $\omega$. We are first going to construct a suitable approximating sequence of generalized indefinite strings $(L_n,\omega _n,\upsilon _n)$ from the set ${\mathcal {F}}_0$. For every $n\in {{\mathbb {N}}}$, let $L_n$ be infinite and choose $R_n>n$ such that

$$\begin{aligned} \int _{R_n}^\infty |{\mathsf {w}}(x)-x|^2 dx < \frac{1}{n}. \end{aligned}$$

We can then find a real-valued function ${\mathsf {w}}_n$ on $[0,\infty )$ which is piecewise constant on the interval $[0,R_n]$ with

$$\begin{aligned} \int _0^{R_n} |{\mathsf {w}}_n(x)-{\mathsf {w}}(x)|^2 dx < \frac{1}{n} \end{aligned}$$

and satisfies $${\mathsf {w}}_n(x)=x$$ for all $$x>R_n$$. The distribution $$\omega _n$$ is now defined in such a way that the corresponding normalized anti-derivative coincides with $${\mathsf {w}}_n$$ almost everywhere. Apart from this, we are able to find a non-negative Borel measure $$\upsilon _n$$ which is supported on a finite set contained in $$[0,R_n)$$ with

$$\begin{aligned} \int _{[0,\infty )} d\upsilon _n = \int _{[0,\infty )} d\upsilon \end{aligned}$$

and such that

$$\begin{aligned} \int _{[0,x)} d\upsilon _n \rightarrow \int _{[0,x)} d\upsilon , \qquad n\rightarrow \infty , \end{aligned}$$

for almost every $x\in [0,\infty )$.

Note that by construction, we then have

5.23 $$\begin{aligned} \int _0^\infty |{\mathsf {w}}_n(x)-x|^2 dx + \int _{[0,\infty )} d\upsilon _n \rightarrow \int _0^\infty |{\mathsf {w}}(x)-x|^2 dx + \int _{[0,\infty )} d\upsilon \end{aligned}$$

as $n\rightarrow \infty$. Furthermore, it follows readily from [20, Proposition 6.2] that the corresponding Weyl–Titchmarsh functions $m_n$ converge locally uniformly to *m*. Thus the associated spectral measures $\mu _n$ certainly satisfy

5.24 $$\begin{aligned} \int _{{\mathbb {R}}}g(\lambda )d\mu _n(\lambda ) \rightarrow \int _{{\mathbb {R}}}g(\lambda ) d\mu (\lambda ), \qquad n\rightarrow \infty , \end{aligned}$$

for every continuous function *g* on ${{\mathbb {R}}}$ with compact support.

In order to prove that the essential spectrum of *S* is restricted to $[0,\infty )$, let *I* be a compact interval in $(-\infty ,0)$. Because of the estimate in Corollary 5.3 and the convergence in (5.23), we see that there is an integer $K_I$ such that $(L_n,\omega _n,\upsilon _n)$ has at most $K_I$ eigenvalues in the interval *I* for every $n\in {{\mathbb {N}}}$. It now follows from the convergence of the measures $\mu _n$ in (5.24) that the limit measure $\mu$ is supported on a finite set on *I*, which implies that *S* has at most finitely many eigenvalues in *I*. Since the interval *I* was arbitrary, we conclude that the essential spectrum of *S* is necessarily contained in $[0,\infty )$.

Now take a compact set $\Omega \subset (0,\infty )$ of positive Lebesgue measure. Due to the convergence of the measures $\mu _n$ in (5.24), we have (see [2, Theorem 30.2])

$$\begin{aligned} \mu (\Omega ) \ge \limsup _{n\rightarrow \infty } \mu _n(\Omega ) = \limsup _{n\rightarrow \infty } \int _\Omega \varrho _n(\lambda )d\lambda , \end{aligned}$$

where the functions $\varrho _n$ are given as in (5.21). An application of Jensen’s inequality [39, Theorem 3.3] then furthermore yields

$$\begin{aligned} \mu (\Omega )&\ge \limsup _{n\rightarrow \infty } D_\Omega \exp \biggl \{\frac{1}{C_\Omega D_\Omega } \int _\Omega \log \biggl (\varrho _n(\lambda )\frac{C_\Omega \lambda ^3}{\sqrt{\lambda }}\biggr ) \frac{\sqrt{\lambda }}{\lambda ^3} d\lambda \biggr \}, \end{aligned}$$

where $C_\Omega$, $D_\Omega$ are positive constants defined as in Corollary 5.4 and by

$$\begin{aligned} D_\Omega = \frac{1}{C_\Omega } \int _\Omega \frac{\sqrt{\lambda }}{\lambda ^3} d\lambda . \end{aligned}$$

In view of the estimate in Corollary 5.4 and the convergence in (5.23), we can conclude that $\mu (\Omega )$ is indeed positive with

$$\begin{aligned} \mu (\Omega )&\ge D_\Omega \exp \biggl \{\frac{-\pi }{C_\Omega D_\Omega } \biggl (\int _0^\infty |{\mathsf {w}}(x)-x|^2dx + \int _{[0,\infty )}d\upsilon \biggr )\biggr \}. \end{aligned}$$

Since all Borel measures on ${{\mathbb {R}}}$ are regular, this readily implies that $\mu (\Omega )$ is positive for every Borel set $\Omega \subseteq [0,\infty )$ of positive Lebesgue measure. With this fact, we have finally verified that the essential spectrum of *S* coincides with the interval $[0,\infty )$ and the absolutely continuous spectrum of *S* is essentially supported on $[0,\infty )$.

In order to finish the proof of Theorem 3.1, let us suppose that *S* is a generalized indefinite string $(L,\omega ,\upsilon )$ such that *L* is infinite and (3.5) holds for a real constant *c* and a positive constant $\eta$. We consider the generalized indefinite string $({L},{\tilde{\omega }},{\tilde{\upsilon }})$, where ${\tilde{\omega }}$ is defined via its normalized anti-derivative ${\tilde{{\mathsf {w}}}}$ by

5.25 $$\begin{aligned} {\tilde{{\mathsf {w}}}}&= \frac{{\mathsf {w}}- c}{\eta } \end{aligned}$$

and ${\tilde{\upsilon }} = \eta ^{-2} \upsilon$. Since $(L,{\tilde{\omega }},{\tilde{\upsilon }})$ satisfies the assumptions imposed before, we infer that the essential spectrum of $(L,{\tilde{\omega }},{\tilde{\upsilon }})$ coincides with the interval $[0,\infty )$ and its absolutely continuous spectrum is essentially supported on $[0,\infty )$. However, as the corresponding Weyl–Titchmarsh functions *m* and ${\tilde{m}}$ are related via

5.26 $$\begin{aligned} m(z) = \eta \, {\tilde{m}}(\eta z) + c, \quad z\in {\mathbb {C}}\backslash {{\mathbb {R}}}, \end{aligned}$$

the same is true for *S*. $\square$

### Remark 5.5

It is natural to ask whether Theorem 3.1 can be deduced from the results of Deift–Killip [14] (or analogous results for one-dimensional Schrödinger operators as in [40]) or Bessonov–Denisov [5], or vice versa. The answer seems to be negative as the distinct forms of the corresponding trace formulas indicate. More precisely, comparing the left-hand side in (5.14) with the corresponding trace formula for one-dimensional Schrödinger operators and the Szegő condition for canonical systems, one can notice that (5.14) requires a rather strong degeneration of the function *a* near zero, but allows for a weaker decay at infinity.

## Proof of Theorem 3.2

In order to prepare for the proof of our second main result, let us fix a positive constant $\alpha$. We assume that $(L,\omega ,\upsilon )$ is a generalized indefinite string such that *L* is infinite and there is an $R>0$ such that the normalized anti-derivative ${\mathsf {w}}$ of $\omega$ satisfies

6.1 $$\begin{aligned} {\mathsf {w}}(x) = \frac{x}{1+2\sqrt{\alpha }x} \end{aligned}$$

for almost all *x* in $[R,\infty )$ and the measure $\upsilon$ vanishes on $[R,\infty )$. In addition, let us also suppose that ${\mathsf {w}}$ is equal to a piecewise constant function almost everywhere on the interval [0, *R*] and that the support of the measure $\upsilon$ is a finite set. For easy reference later on, we will denote the set of generalized indefinite strings defined in this way by ${\mathcal {F}}_\alpha$. Under these assumptions, for every $k\in {\mathbb {C}}_+$, there is a *Jost solution* $f(k,\cdot \,)$ of the differential equation (2.8) with $z=k^2+\alpha \in {\mathbb {C}}\backslash [\alpha ,\infty )$ such that

6.2 $$\begin{aligned} f(k,x) = (1+2\sqrt{\alpha }x)^{\frac{\mathrm {i}k}{2\sqrt{\alpha }} + \frac{1}{2}}, \quad x\in [R,\infty ). \end{aligned}$$

We note that since the function $f(k,\cdot \,)$ clearly lies in ${\dot{H}}^1[0,\infty )$ and $L^2([0,\infty );\upsilon )$, the corresponding Weyl–Titchmarsh function *m* is given by

6.3 $$\begin{aligned} m(k^2+\alpha ) = \frac{f'(k,0-)}{(k^2+\alpha )f(k,0)} \end{aligned}$$

as long as $k^2+\alpha \in {\mathbb {C}}\backslash {{\mathbb {R}}}$. Furthermore, we define the function *a* on ${\mathbb {C}}_+$ via

6.4 $$\begin{aligned} a(k) = \frac{(\mathrm {i}k - \sqrt{\alpha })f(k,0)+f'(k,0-)}{2\mathrm {i}k}, \quad k\in {\mathbb {C}}_+. \end{aligned}$$

If we denote with $\theta$, $\phi$ the fundamental system of solutions of the differential equation (2.8) as in Sect. 4, then we readily compute that

6.5 $$\begin{aligned} \begin{aligned} f(k,0)&= (1+2\sqrt{\alpha }R)^{\frac{\mathrm {i}k}{2\sqrt{\alpha }}+\frac{1}{2}} \phi ^{[1]}(k^2+\alpha ,R) \\&\quad - (\mathrm {i}k+\sqrt{\alpha })(1-(\mathrm {i}k-\sqrt{\alpha })R)(1+2\sqrt{\alpha }R)^{\frac{\mathrm {i}k}{2\sqrt{\alpha }}-\frac{1}{2}} \phi (k^2+\alpha ,R), \end{aligned} \end{aligned}$$

6.6 $$\begin{aligned} \begin{aligned} f'(k,0-)&= - (1+2\sqrt{\alpha }R)^{\frac{\mathrm {i}k}{2\sqrt{\alpha }}+\frac{1}{2}} \theta ^{[1]}(k^2+\alpha ,R) \\&\qquad + (\mathrm {i}k+\sqrt{\alpha })(1-(\mathrm {i}k-\sqrt{\alpha })R)(1+2\sqrt{\alpha }R)^{\frac{\mathrm {i}k}{2\sqrt{\alpha }}-\frac{1}{2}} \theta (k^2+\alpha ,R), \end{aligned} \end{aligned}$$

which yields, upon plugging these expressions into the definition of *a*, that

6.7 $$\begin{aligned} \begin{aligned}&\frac{2\mathrm {i}k}{(1+2\sqrt{\alpha }R)^{\frac{\mathrm {i}k}{2\sqrt{\alpha }} + \frac{1}{2}}}a(k) =\frac{(\mathrm {i}k + \sqrt{\alpha })(1 - (\mathrm {i}k - \sqrt{\alpha })R)}{1+2\sqrt{\alpha }R}\theta (k^2+\alpha ,R) - \theta ^{[1]}(k^2+\alpha ,R) \\&\qquad \quad + \frac{1-(\mathrm {i}k - \sqrt{\alpha })R}{1+2\sqrt{\alpha }R}(k^2+\alpha )\phi (k^2+\alpha ,R) + (\mathrm {i}k - \sqrt{\alpha })\phi ^{[1]}(k^2+\alpha ,R) \end{aligned}\end{aligned}$$

for all $k\in {\mathbb {C}}_+$. Due to our assumptions on the supports of $\omega$ and $\upsilon$ on [0, *R*], the right-hand side of this equation turns out to be a polynomial in *k*. This guarantees that the function *a* admits an analytic continuation (denoted with *a* as well for simplicity) to all of ${\mathbb {C}}$ except for zero, where *a* has at most a simple pole. It furthermore entails that *a* has only finitely many zeros, none of which lie on the real axis. In order to prove this, we introduce the function *b* on ${\mathbb {C}}_+$ next via

6.8 $$\begin{aligned} b(k) = \frac{(\mathrm {i}k + \sqrt{\alpha })f(k,0) - f'(k,0-)}{2\mathrm {i}k}, \quad k\in {\mathbb {C}}_+. \end{aligned}$$

One infers from the expressions in (6.5) and (6.6) that *b* also admits an analytic continuation to all of ${\mathbb {C}}$ except for zero. Now after a straightforward computation, we see that for all non-zero real *k*, we have

6.9 $$\begin{aligned} a(k)^*&= a(-k),&b(k)^*&= b(-k), \end{aligned}$$

as well as

6.10 $$\begin{aligned} |a(k)|^2 = |b(k)|^2 + 1, \end{aligned}$$

which guarantees that *a* has no zeros on the real axis. Moreover, all zeros in ${\mathbb {C}}_+$ necessarily have to lie on the imaginary axis. In fact, if *k* was a zero of *a* in ${\mathbb {C}}_+$ that does not lie on the imaginary axis, then $k^2+\alpha \in {\mathbb {C}}\backslash {{\mathbb {R}}}$ and (6.4) would imply

6.11 $$\begin{aligned} f'(k,0-) = -(\mathrm {i}k - \sqrt{\alpha })f(k,0). \end{aligned}$$

By using (6.3), this would allow us to compute the imaginary part

6.12 $$\begin{aligned} \begin{aligned} \mathrm {Im}\, m(k^2+\alpha )&= \mathrm {Im}\, \frac{f'(k,0-)}{(k^2+\alpha ) f(k,0)} = \mathrm {Im}\, \frac{1}{\mathrm {i}k+\sqrt{\alpha }} = - \frac{\mathrm {Re}\, k}{|\mathrm {i}k+\sqrt{\alpha }|^2} \\&= - \frac{\mathrm {Im}\, (k^2+\alpha )}{2\mathrm {Im}\, k\,|\mathrm {i}k+\sqrt{\alpha }|^2}, \end{aligned}\end{aligned}$$

which is a contradiction to the fact that *m* is a Herglotz–Nevanlinna function. As this proves that all zeros in ${\mathbb {C}}_+$ indeed lie on the imaginary axis, we may enumerate them, repeated according to multiplicity, by $\mathrm {i}\kappa _1,\ldots ,\mathrm {i}\kappa _N$ for some positive constants $\kappa _1,\ldots ,\kappa _N$. The next result collects two trace formulas which are going to play a key role in the proof of Theorem 3.2.

### Lemma 6.1

If the generalized indefinite string $(L,\omega ,\upsilon )$ belongs to ${\mathcal {F}}_\alpha$, then we have the identities

6.13 $$\begin{aligned} \begin{aligned}&\frac{1}{\sqrt{\alpha }}\sum _{n=1}^N \frac{ \kappa _n}{\kappa _n^2-\alpha } + \frac{1}{2{\alpha }}\sum _{n=1}^N\log \left| \frac{\kappa _n - \sqrt{\alpha }}{\kappa _n + \sqrt{\alpha }}\right| + \frac{\sqrt{\alpha }}{\pi } \int _{{\mathbb {R}}}\frac{1}{(k^2+{\alpha })^2} \log |a(k)|dk \\&\qquad = \int _0^\infty {\mathsf {w}}(x) - \frac{x}{1+2\sqrt{\alpha }x}\,dx \end{aligned}\end{aligned}$$

and

6.14 $$\begin{aligned} \begin{aligned}&\frac{1}{2{\alpha }}\sum _{n=1}^N \frac{\kappa _n^3+\alpha \kappa _n }{(\kappa _n^2-\alpha )^2} + \frac{1}{4{\alpha }^{3/2}}\sum _{n=1}^N\log \left| \frac{\kappa _n - \sqrt{\alpha }}{\kappa _n + \sqrt{\alpha }}\right| + \frac{2}{\pi } \int _{{\mathbb {R}}}\frac{k^2}{(k^2+{\alpha })^3} \log |a(k)|dk \\&\qquad =\int _0^\infty \Bigl |{\mathsf {w}}(x) - \frac{x}{1+2\sqrt{\alpha }x} \Bigr |^2 (1+2\sqrt{\alpha }x) dx + \int _{[0,\infty )} (1+2\sqrt{\alpha }x) d\upsilon (x). \end{aligned}\end{aligned}$$

### Proof

It follows readily from (6.7) and (4.6) that

$$\begin{aligned} a(\mathrm {i}\sqrt{\alpha }) = 1. \end{aligned}$$

By differentiating both sides in (6.7), evaluating at $\mathrm {i}\sqrt{\alpha }$ and using the expressions from Proposition 4.1, we also see that

$$\begin{aligned} a'(\mathrm {i}\sqrt{\alpha })&= 2\mathrm {i}\sqrt{\alpha }\int _0^\infty {\mathsf {w}}(x) - {\mathsf {w}}_{\alpha }(x)\,dx, \end{aligned}$$

where the function ${\mathsf {w}}_\alpha$ is simply given by

$$\begin{aligned} {\mathsf {w}}_\alpha (x) = \frac{x}{1+2\sqrt{\alpha }x}, \quad x\in [0,\infty ), \end{aligned}$$

as in Example B. After similar but longer computations, we furthermore get

$$\begin{aligned} \begin{aligned} a''(\mathrm {i}\sqrt{\alpha })&= 4{\sqrt{\alpha }} \int _0^\infty ({\mathsf {w}}(x)-{\mathsf {w}}_{\alpha }(x))^2 (1+2\sqrt{\alpha }x) dx\\&\quad + 4{\sqrt{\alpha }} \int _{[0,\infty )} (1+2\sqrt{\alpha }x) d\upsilon (x) \\&\quad - 4\alpha \biggl (\int _0^\infty {\mathsf {w}}(x) - {\mathsf {w}}_{\alpha }(x)\, dx \biggr )^2 - 2 \int _0^\infty {\mathsf {w}}(x) - {\mathsf {w}}_{\alpha }(x)\, dx. \end{aligned} \end{aligned}$$

Since the function *a* is of bounded type in the upper complex half-plane, it admits a Nevanlinna factorization [38, Theorem 6.13] of the form

6.15 $$\begin{aligned} a(k) = C \prod _{n=1}^N \frac{\mathrm {i}\kappa _n - k}{\mathrm {i}\kappa _n + k} \exp \biggl \{-\mathrm {i}\beta k + \frac{\mathrm {i}\gamma }{k} + \frac{1}{\pi \mathrm {i}} \int _{{\mathbb {R}}}\biggl (\frac{1}{t-k}-\frac{t}{1+t^2}\biggr )\log |a(t)|dt \biggr \} \end{aligned}$$

for all $k\in {\mathbb {C}}_+$, where $\beta$ and $\gamma$ are real constants and *C* is a complex constant with modulus one. However, as zero is at most a simple pole of *a*, we infer that the constant $\gamma$ has to be equal to zero.

After differentiating (6.15), we obtain

6.16 $$\begin{aligned} \frac{a'(k)}{a(k)} = \sum _{n=1}^N \frac{2\mathrm {i}\kappa _n}{\kappa _n^2+k^2} -\mathrm {i}\beta + \frac{1}{\pi \mathrm {i}} \int _{{\mathbb {R}}}\frac{1}{(t-k)^2} \log |a(t)|dt \end{aligned}$$

for all $k\in {\mathbb {C}}_+$ close enough to $\mathrm {i}\sqrt{\alpha }$ (so that *a*(*k*) is non-zero). Upon differentiating one more time, we also get

6.17 $$\begin{aligned} \frac{a''(k)}{a(k)} - \frac{a'(k)^2}{a(k)^2} = \sum _{n=1}^N \frac{ - 4\mathrm {i}\kappa _n k}{(\kappa _n^2+k^2)^2} + \frac{2}{\pi \mathrm {i}} \int _{{\mathbb {R}}}\frac{1}{(t-k)^3} \log |a(t)|dt, \end{aligned}$$

again, as long as $k\in {\mathbb {C}}_+$ is close enough to $\mathrm {i}\sqrt{\alpha }$.

Now we evaluate both sides of (6.15) at $\mathrm {i}\sqrt{\alpha }$ and take absolute values to obtain

$$\begin{aligned} 1 = \prod _{n=1}^N \left| \frac{\kappa _n - \sqrt{\alpha }}{\kappa _n + \sqrt{\alpha }}\right| \exp \biggl \{ \beta \sqrt{\alpha } + \frac{\sqrt{\alpha }}{\pi } \int _{{\mathbb {R}}}\frac{1}{t^2+{\alpha }}\log |a(t)|dt\biggr \}. \end{aligned}$$

This gives the following expression for $\beta$ in terms of the scattering data:

$$\begin{aligned} \beta = \frac{1}{\sqrt{\alpha }}\sum _{n=1}^N\log \left| \frac{\kappa _n + \sqrt{\alpha }}{\kappa _n - \sqrt{\alpha }}\right| - \frac{1}{\pi } \int _{{\mathbb {R}}}\frac{1}{t^2+{\alpha }}\log |a(t)|dt. \end{aligned}$$

By evaluating equality (6.16) at $\mathrm {i}\sqrt{\alpha }$, we next get

$$\begin{aligned} 2\mathrm {i}\sqrt{\alpha }\int _0^\infty {\mathsf {w}}(x) - {\mathsf {w}}_{\alpha }(x)\,dx =&\sum _{n=1}^N \frac{2\mathrm {i}\kappa _n}{\kappa _n^2-\alpha } -\mathrm {i}\beta + \frac{1}{\pi \mathrm {i}} \int _{{\mathbb {R}}}\frac{1}{(t-\mathrm {i}\sqrt{\alpha })^2} \log |a(t)|dt. \end{aligned}$$

Taking imaginary parts and using the expression for $\beta$ yields (6.13). In a similar manner, the real part of the right-hand side of (6.17) evaluated at $\mathrm {i}\sqrt{\alpha }$ becomes

$$\begin{aligned} \sum _{n=1}^N \frac{4\sqrt{\alpha } \kappa _n }{(\kappa _n^2-\alpha )^2} + \frac{2}{\pi } \int _{{\mathbb {R}}}\frac{3t^2\sqrt{\alpha } - \alpha ^{3/2}}{(t^2+{\alpha })^3} \log |a(t)|dt \end{aligned}$$

and the left-hand side

$$\begin{aligned}&4{\sqrt{\alpha }} \int _0^\infty ({\mathsf {w}}(x)-{\mathsf {w}}_{\alpha }(x))^2 (1+2\sqrt{\alpha }x) dx + 4{\sqrt{\alpha }} \int _{[0,\infty )} (1+2\sqrt{\alpha }x) d\upsilon (x) \\&\qquad - \frac{2}{\sqrt{\alpha }}\sum _{n=1}^N \frac{ \kappa _n}{\kappa _n^2-\alpha } - \frac{1}{\alpha }\sum _{n=1}^N\log \left| \frac{\kappa _n - \sqrt{\alpha }}{\kappa _n + \sqrt{\alpha }}\right| - \frac{2\sqrt{\alpha }}{\pi } \int _{{\mathbb {R}}}\frac{1}{(t^2+{\alpha })^2} \log |a(t)|dt, \end{aligned}$$

where we also made use of equality (6.13). After equating these two expressions, we readily end up with (6.14). $\square$

We note that the integral term on the left-hand side of the identity (6.14) is non-negative in view of (6.10). In particular, this observation will allow us to obtain a Lieb–Thirring-type estimate on the eigenvalues of the corresponding self-adjoint realization below $\alpha$. To this end, we first point out that there are only finitely many such eigenvalues. More precisely, we see from (6.5) and (6.6) that the right-hand side of (6.3) is a rational function. This implies that the Weyl–Titchmarsh function *m* has a continuation to a meromorphic function on ${\mathbb {C}}\backslash [\alpha ,\infty )$ with only finitely many poles, none of which is located at zero. Hence, we may enumerate all eigenvalues below $\alpha$ in the following way:

6.18 $$\begin{aligned} \lambda _{K_-}^-< \dots< \lambda _{1}^-< 0< \lambda _{1}^+<\dots< \lambda _{K_+}^+ <\alpha . \end{aligned}$$

### Corollary 6.2

If the generalized indefinite string $(L,\omega ,\upsilon )$ belongs to ${\mathcal {F}}_\alpha$, then we have the estimate

6.19 $$\begin{aligned} \begin{aligned}&\frac{4}{3 \alpha ^{3/2}}\sum _{i=1}^{K_-} \biggl (1-\frac{\lambda _i^-}{{\alpha }}\biggr )^{-3/2} + \frac{4}{3\alpha ^{3/2}}\sum _{i=1}^{K_+} \biggl (1-\frac{\lambda _i^+}{{\alpha }}\biggr )^{3/2} \\&\qquad \le \int _0^\infty \Bigl |{\mathsf {w}}(x) - \frac{x}{1+2\sqrt{\alpha }x} \Bigr |^2 (1+2\sqrt{\alpha }x) dx + \int _{[0,\infty )} (1+2\sqrt{\alpha }x) d\upsilon (x). \end{aligned}\end{aligned}$$

### Proof

Since the function *m* is a Herglotz–Nevanlinna function, we see from (6.3) that the function

$$\begin{aligned} \kappa \mapsto \frac{f'(\mathrm {i}\kappa ,0-)}{(-\kappa ^2+\alpha ) f(\mathrm {i}\kappa ,0)} \end{aligned}$$

is real-valued, continuous and strictly decreasing for positive $\kappa$ away from the poles

$$\begin{aligned} \sqrt{\alpha -\lambda _{K_+}^+},\ldots ,\sqrt{\alpha -\lambda _{1}^+},\sqrt{\alpha -\lambda _1^-},\ldots ,\sqrt{\alpha -\lambda _{K_-}^-}. \end{aligned}$$

Because of this, we can find a positive $\kappa$ such that

$$\begin{aligned} \frac{f'(\mathrm {i}\kappa ,0-)}{(-\kappa ^2+\alpha ) f(\mathrm {i}\kappa ,0)} = \frac{1}{\sqrt{\alpha }-\kappa } \end{aligned}$$

between each pair of consecutive points in the sequence

$$\begin{aligned} \sqrt{\alpha -\lambda _{K_+}^+},\ldots ,\sqrt{\alpha -\lambda _{1}^+},\sqrt{\alpha },\sqrt{\alpha -\lambda _1^-},\ldots ,\sqrt{\alpha -\lambda _{K_-}^-}. \end{aligned}$$

As $\mathrm {i}\kappa$ is a zero of the function *a* for each such $\kappa$, we conclude that

$$\begin{aligned} \sqrt{\alpha -\lambda _{K_+}^+}<\kappa _{n_+(K_+)}<\sqrt{\alpha -\lambda _{K_+-1}^+}< \cdots< \sqrt{\alpha -\lambda _{1}^+}< \kappa _{n_+(1)} < \sqrt{\alpha } \end{aligned}$$

as well as

$$\begin{aligned} \sqrt{\alpha }< \kappa _{n_-(1)}< \sqrt{\alpha -\lambda _1^-}<\cdots<\sqrt{\alpha -\lambda _{K_--1}^-}< \kappa _{n_-(K_-)} < \sqrt{\alpha -\lambda _{K_-}^-} \end{aligned}$$

for some indices $n_+(K_+),\ldots ,n_+(1),n_-(1),\ldots ,n_-(K_-)\in \{1,\ldots ,N\}$.

Now let us consider the function *F* defined by

6.20 $$\begin{aligned} F(s) = {2} \frac{s^3+s}{(s^2-1)^2} + \log \left| \frac{s - 1}{s + 1}\right| , \quad s\in (0,1)\cup (1,\infty ), \end{aligned}$$

and first notice that

$$\begin{aligned} F(1/s) = F(s), \quad s\in (0,1)\cup (1,\infty ). \end{aligned}$$

It is also straightforward to see that *F* is strictly increasing on (0, 1) and strictly decreasing on $(1,\infty )$ since we may compute

$$\begin{aligned} F'(s) = -\frac{16s^2}{(s^2-1)^3}, \quad s\in (0,1)\cup (1,\infty ). \end{aligned}$$

Moreover, the function *F* satisfies the bound

$$\begin{aligned} F(s) = \int _s^\infty \frac{16 r^2}{(r^2-1)^3} dr \ge \int _s^\infty \frac{16}{r^4} dr = \frac{16}{3 s^3} > 0, \quad s\in (1,\infty ). \end{aligned}$$

By combining all these facts, we can estimate

$$\begin{aligned}&\biggl (1-\frac{\lambda _i^\pm }{{\alpha }}\biggr )^{\pm 3/2} < \biggl (\frac{\kappa _{n_\pm (i)}}{\sqrt{\alpha }}\biggr )^{\pm 3} \le \frac{3}{16} F\biggl (\frac{\kappa _{n_\pm (i)}}{\sqrt{\alpha }}\biggr ) \end{aligned}$$

for all $i\in \{1,\ldots ,K_\pm \}$. This allows us to bound the left-hand side of (6.19) by

$$\begin{aligned} \frac{1}{4\alpha ^{3/2}} \sum _{i=1}^{K_-} F\biggl (\frac{\kappa _{n_-(i)}}{\sqrt{\alpha }}\biggr ) + \frac{1}{4\alpha ^{3/2}} \sum _{i=1}^{K_+} F\biggl (\frac{\kappa _{n_+(i)}}{\sqrt{\alpha }}\biggr ) \le \frac{1}{4\alpha ^{3/2}} \sum _{n=1}^{N} F\biggl (\frac{\kappa _{n}}{\sqrt{\alpha }}\biggr ). \end{aligned}$$

It is readily seen that this sum coincides with the first two terms in (6.14), which yields the claim as the integral term on the left-hand side there is non-negative. $\square$

We are now going to use the identity (6.14) to estimate the absolutely continuous spectrum of $(L,\omega ,\upsilon )$. To this end, we first note that we have

6.21 $$\begin{aligned} (k^2+\alpha )m(k^2+\alpha ) = \sqrt{\alpha }-\mathrm {i}k\,\frac{b(k)-a(k)}{b(k) + a(k)} \end{aligned}$$

for all $k\in {\mathbb {C}}_+$ with $k^2+\alpha \in {\mathbb {C}}\backslash {{\mathbb {R}}}$. Since the functions *a* and *b* are analytic on all of ${\mathbb {C}}$ except for zero and satisfy the properties (6.9) and (6.10) on the real line, one can conclude that the spectrum of $(L,\omega ,\upsilon )$ on the interval $(\alpha ,\infty )$ is purely absolutely continuous with the corresponding spectral measure $\mu$ given by

6.22 $$\begin{aligned} \mu (B) = \int _B \varrho (\lambda ) d\lambda \end{aligned}$$

for every Borel set $B\subseteq (\alpha ,\infty )$, where $\varrho$ is defined by

6.23 $$\begin{aligned} \varrho (\lambda ) = \lim _{\varepsilon \rightarrow 0} \frac{1}{\pi }\mathrm {Im}\, m(\lambda +\mathrm {i}\varepsilon ) = \frac{\sqrt{\lambda -\alpha }}{\pi \lambda |b(\sqrt{\lambda - \alpha }) + a(\sqrt{\lambda - \alpha })|^2}, \quad \lambda \in (\alpha ,\infty ). \end{aligned}$$

We note that the function $\varrho$ is continuous and positive on $(\alpha ,\infty )$.

### Corollary 6.3

If the generalized indefinite string $(L,\omega ,\upsilon )$ belongs to ${\mathcal {F}}_\alpha$, then for every compact subset $\Omega$ of $(\alpha ,\infty )$ we have the estimate

6.24 $$\begin{aligned} \begin{aligned}&- \frac{1}{\pi }\int _\Omega \log \biggl (\varrho (\lambda ) \frac{4\pi \lambda ^3}{\alpha ^2\sqrt{\lambda -\alpha }}\biggl ) \frac{\sqrt{\lambda -\alpha }}{\lambda ^3} d\lambda \\&\qquad \le \int _0^\infty \Bigl |{\mathsf {w}}(x) - \frac{x}{1+2\sqrt{\alpha }x} \Bigr |^2 (1+2\sqrt{\alpha }x) dx + \int _{[0,\infty )} (1+2\sqrt{\alpha }x) d\upsilon (x). \end{aligned} \end{aligned}$$

### Proof

For every positive *k*, we first compute that

$$\begin{aligned} \left| 1- \frac{b(k)-a(k)}{b(k)+a(k)}\right| ^2 = \frac{4 |a(k)|^2}{|b(k)+a(k)|^2} = 4\pi \frac{k^2+\alpha }{k} \varrho (k^2+\alpha ) |a(k)|^2 \end{aligned}$$

and on the other side that

$$\begin{aligned} \left| 1- \frac{b(k)-a(k)}{b(k)+a(k)}\right| \ge \mathrm {Re}\left( 1- \frac{b(k)-a(k)}{b(k)+a(k)}\right) = 1+\frac{1}{|b(k)+a(k)|^2} \ge 1. \end{aligned}$$

In combination, this gives the bound

$$\begin{aligned} \frac{1}{|a(\sqrt{\lambda -\alpha })|^2} \le \frac{4\pi \lambda }{\sqrt{\lambda -\alpha }} \varrho (\lambda ) \le \frac{4\pi \lambda ^3}{\alpha ^2\sqrt{\lambda -\alpha }} \varrho (\lambda ) \end{aligned}$$

as long as $\lambda \in \Omega$, which allows us to estimate the integral

$$\begin{aligned} - \frac{1}{\pi } \int _\Omega \log \biggl (\varrho (\lambda ) \frac{4\pi \lambda ^3}{\alpha ^2\sqrt{\lambda -\alpha }}\biggl ) \frac{\sqrt{\lambda -\alpha }}{\lambda ^3} d\lambda \le \frac{1}{\pi } \int _\Omega \log |a(\sqrt{\lambda -\alpha })|^2 \frac{\sqrt{\lambda -\alpha }}{\lambda ^3}d\lambda . \end{aligned}$$

Upon employing a substitution, we can further bound this by

$$\begin{aligned} \frac{2}{\pi } \int _{\sqrt{\min \Omega -\alpha }}^{\sqrt{\max \Omega -\alpha }} \log |a(k)|^2 \frac{k^2}{(k^2+\alpha )^3} dk \le \frac{2}{\pi } \int _{{{\mathbb {R}}}} \log |a(k)| \frac{k^2}{(k^2+\alpha )^3} dk. \end{aligned}$$

Now it remains to notice that the sum of the first two terms in (6.14) is non-negative since the function *F* defined in (6.20) takes positive values. $\square$

We are now ready to prove our second main result.

### Proof of Theorem 3.2

Let us assume for now that *S* is a generalized indefinite string $(L,\omega ,\upsilon )$ such that *L* is infinite and

$$\begin{aligned} \int _0^\infty \Bigl | {\mathsf {w}}(x) - \frac{x}{1+2\sqrt{\alpha }x}\Bigr |^2 x\, dx + \int _{[0,\infty )} x\, d\upsilon (x)&< \infty , \end{aligned}$$

where ${\mathsf {w}}$ is the normalized anti-derivative of $\omega$. We are first going to construct a suitable approximating sequence of generalized indefinite strings $(L_n,\omega _n,\upsilon _n)$ from the set ${\mathcal {F}}_\alpha$. For every $n\in {{\mathbb {N}}}$, let $L_n$ be infinite and choose $R_n>n$ such that

$$\begin{aligned} \int _{R_n}^\infty \Bigl |{\mathsf {w}}(x)-\frac{x}{1+2\sqrt{\alpha }x}\Bigr |^2 x\, dx < \frac{1}{n}. \end{aligned}$$

We can then find a real-valued function ${\mathsf {w}}_n$ on $[0,\infty )$ which is piecewise constant on the interval $[0,R_n]$ with

$$\begin{aligned} \int _0^{R_n} |{\mathsf {w}}_n(x)-{\mathsf {w}}(x)|^2 dx&< \frac{1}{n R_n} \end{aligned}$$

and which is given explicitly by

$$\begin{aligned} {\mathsf {w}}_n(x)&= \frac{x}{1+2\sqrt{\alpha }x} \end{aligned}$$

for all $$x>R_n$$. The distribution $$\omega _n$$ is now defined in such a way that the corresponding normalized anti-derivative coincides with $${\mathsf {w}}_n$$ almost everywhere. Apart from this, we are able to find a non-negative Borel measure $$\upsilon _n$$ which is supported on a finite set contained in $$[0,R_n)$$ with

$$\begin{aligned} \int _{[0,\infty )} d\upsilon _n&= \int _{[0,\infty )} d\upsilon ,&\int _{[0,\infty )} x\, d\upsilon _n(x)&\le \int _{[0,\infty )} x\, d\upsilon (x), \end{aligned}$$

and such that for almost every $x\in [0,\infty )$ we have

$$\begin{aligned} \int _{[0,x)} d\upsilon _n \rightarrow \int _{[0,x)} d\upsilon , \qquad n\rightarrow \infty . \end{aligned}$$

Note that by construction, there is a positive constant *M* such that

6.25 $$\begin{aligned} \int _0^\infty \Bigl |{\mathsf {w}}_n(x)-\frac{x}{1+2\sqrt{\alpha }x}\Bigr |^2 (1+2\sqrt{\alpha }x) dx + \int _{[0,\infty )} (1+2\sqrt{\alpha }x) d\upsilon _n(x) \le M \end{aligned}$$

for all $n\in {{\mathbb {N}}}$. Furthermore, it follows readily from [20, Proposition 6.2] that the corresponding Weyl–Titchmarsh functions $m_n$ converge locally uniformly to *m*. Thus the associated spectral measures $\mu _n$ certainly satisfy

6.26 $$\begin{aligned} \int _{{\mathbb {R}}}g(\lambda )d\mu _n(\lambda ) \rightarrow \int _{{\mathbb {R}}}g(\lambda ) d\mu (\lambda ), \qquad n\rightarrow \infty , \end{aligned}$$

for every continuous function *g* on ${{\mathbb {R}}}$ with compact support.

In order to prove that the essential spectrum of *S* is restricted to $[\alpha ,\infty )$, let *I* be a compact interval in $(-\infty ,\alpha )$. Because of the estimate in Corollary 6.2 and the bound in (6.25), we see that there is an integer $K_I$ such that $(L_n,\omega _n,\upsilon _n)$ has at most $K_I$ eigenvalues in the interval *I* for every $n\in {{\mathbb {N}}}$. It now follows from the convergence of the measures $\mu _n$ in (6.26) that the limit measure $\mu$ is supported on a finite set on *I*, which implies that *S* has at most finitely many eigenvalues in *I*. Since the interval *I* was arbitrary, we conclude that the essential spectrum of *S* is necessarily contained in $[\alpha ,\infty )$.

Now take a compact set $\Omega \subset (\alpha ,\infty )$ of positive Lebesgue measure. Due to the convergence of the measures $\mu _n$ in (6.26), we have (see [2, Theorem 30.2])

$$\begin{aligned} \mu (\Omega ) \ge \limsup _{n\rightarrow \infty } \mu _n(\Omega ) = \limsup _{n\rightarrow \infty } \int _\Omega \varrho _n(\lambda )d\lambda , \end{aligned}$$

where the functions $\varrho _n$ are given as in (6.23). An application of Jensen’s inequality [39, Theorem 3.3] then furthermore yields

$$\begin{aligned} \mu (\Omega )&\ge \limsup _{n\rightarrow \infty } D_\Omega \exp \biggl \{\frac{\alpha ^2}{4\pi D_\Omega } \int _\Omega \log \biggl (\varrho _n(\lambda )\frac{4\pi \lambda ^3}{\alpha ^2\sqrt{\lambda -\alpha }}\biggr ) \frac{\sqrt{\lambda -\alpha }}{\lambda ^3} d\lambda \biggr \}, \end{aligned}$$

where $D_\Omega$ is a positive constant defined by

$$\begin{aligned} D_\Omega = \int _\Omega \frac{\alpha ^2\sqrt{\lambda -\alpha }}{4\pi \lambda ^3} d\lambda . \end{aligned}$$

In view of the estimate in Corollary 6.3 and the bound in (6.25), we conclude that

$$\begin{aligned} \mu (\Omega )&\ge D_\Omega \mathrm {e}^{\frac{-\alpha ^2 M}{4 D_\Omega }} > 0. \end{aligned}$$

Since all Borel measures on ${{\mathbb {R}}}$ are regular, this readily implies that $\mu (\Omega )$ is positive for every Borel set $\Omega \subseteq [\alpha ,\infty )$ of positive Lebesgue measure. Thus, we have finally verified that the essential spectrum of *S* coincides with the interval $[\alpha ,\infty )$ and the absolutely continuous spectrum of *S* is essentially supported on $[\alpha ,\infty )$.

In order to finish the proof of Theorem 3.2, let us suppose that *S* is a generalized indefinite string $(L,\omega ,\upsilon )$ such that *L* is infinite and (3.10) holds for a real constant *c* and a positive constant $\eta$. We consider the generalized indefinite string $({L},{\tilde{\omega }},{\tilde{\upsilon }})$, where ${\tilde{\omega }}$ is defined via its normalized anti-derivative ${\tilde{{\mathsf {w}}}}$ by (5.25) and ${\tilde{\upsilon }} = \eta ^{-2} \upsilon$. Since $(L,{\tilde{\omega }},{\tilde{\upsilon }})$ satisfies the assumptions imposed before, we infer that the essential spectrum of $(L,{\tilde{\omega }},{\tilde{\upsilon }})$ coincides with the interval $[\alpha ,\infty )$ and its absolutely continuous spectrum is essentially supported on $[\alpha ,\infty )$. However, as the corresponding Weyl–Titchmarsh functions *m* and ${\tilde{m}}$ are related via (5.26), we see that the essential spectrum of *S* coincides with the interval $[\alpha /\eta ,\infty )$ and its absolutely continuous spectrum is essentially supported on $[\alpha /\eta ,\infty )$. $\square$

## The Conservative Camassa–Holm Flow

In this section, we are going to demonstrate how our results apply to the isospectral problem of the conservative Camassa–Holm flow. To this end, let *u* be a real-valued function in $H^1_{\mathrm {loc}}[0,\infty )$ and $\upsilon$ be a non-negative Borel measure on $[0,\infty )$. We define the distribution $\omega$ in $H^{-1}_{\mathrm {loc}}[0,\infty )$ by

7.1 $$\begin{aligned} \omega (h) = \int _0^\infty u(x)h(x)dx + \int _0^\infty u'(x)h'(x)dx, \quad h\in H^1_{\mathrm {c}}[0,\infty ), \end{aligned}$$

so that $\omega = u - u''$ in a distributional sense. Now the isospectral problem of the conservative Camassa–Holm flow is associated with the differential equation

7.2 $$\begin{aligned} -g'' + \frac{1}{4} g = z\, \omega \, g + z^2 \upsilon \, g, \end{aligned}$$

where *z* is a spectral parameter. Just like for generalized indefinite strings, this differential equation has to be understood in a weak sense in general: A solution of (7.2) is a function $g\in H^1_{{\mathrm {loc}}}[0,\infty )$ such that

7.3 $$\begin{aligned} \Delta _g h(0) + \int _{0}^\infty g'(x) h'(x) dx + \frac{1}{4} \int _0^\infty g(x)h(x)dx = z\, \omega (gh) + z^2 \upsilon (g h) \end{aligned}$$

for some constant $\Delta _g\in {\mathbb {C}}$ and every function $h\in H^1_{\mathrm {c}}[0,\infty )$. For such a solution *g*, the constant $\Delta _g$ is uniquely determined and will be denoted with $g'(0-)$.

We are first going to show that it is always possible to transform the differential equation (7.2) into the differential equation

7.4 $$\begin{aligned} - f'' = z\,\omega _\mathrm {s}f + z^2\upsilon _\mathrm {s}f \end{aligned}$$

for some corresponding generalized indefinite string $(\infty ,\omega _\mathrm {s},\upsilon _\mathrm {s})$. To this end, let us introduce the diffeomorphism $\mathrm {s}:[0,\infty )\rightarrow [0,\infty )$ by

7.5 $$\begin{aligned} \mathrm {s}(t) = \log (1+t), \quad t\in [0,\infty ), \end{aligned}$$

and note that the inverse of $\mathrm {s}$ is simply given by

7.6 $$\begin{aligned} t = \mathrm {s}^{-1}(x) = \mathrm {e}^{x} - 1, \quad x\in [0,\infty ). \end{aligned}$$

Next we define a real-valued measurable function ${\mathsf {w}}_\mathrm {s}$ on $[0,\infty )$ such that

7.7 $$\begin{aligned} {\mathsf {w}}_\mathrm {s}(t) = u(0) - \frac{u'(\mathrm {s}(t))+ u(\mathrm {s}(t))}{1+t} \end{aligned}$$

for almost all $t\in [0,\infty )$, where we note that the right-hand side is well-defined almost everywhere. It follows readily that the function ${\mathsf {w}}_\mathrm {s}$ is locally square integrable, so that we can find a real distribution $\omega _\mathrm {s}$ in $H^{-1}_{\mathrm {loc}}[0,\infty )$ which has ${\mathsf {w}}_\mathrm {s}$ as its normalized anti-derivative. Furthermore, the non-negative Borel measure $\upsilon _\mathrm {s}$ on $[0,\infty )$ is given by setting

7.8 $$\begin{aligned} \upsilon _\mathrm {s}(B) = \int _B \frac{1}{1+t}\, d\upsilon \circ \mathrm {s}(t) = \int _{\mathrm {s}(B)} \mathrm {e}^{-x} d\upsilon (x) \end{aligned}$$

for every Borel set $B\subseteq [0,\infty )$. This defines a generalized indefinite string $(\infty ,\omega _\mathrm {s},\upsilon _\mathrm {s})$ whose relation to the differential equation (7.2) we are going to describe now.

### Lemma 7.1

A function *g* is a solution of the differential equation (7.2) if and only if the function *f* defined by

7.9 $$\begin{aligned} f(t) = g(\mathrm {s}(t)) \sqrt{1+t}, \quad t\in [0,\infty ), \end{aligned}$$

is a solution of the differential equation (7.4).

### Proof

Let us suppose that two functions *f* and *g* on $[0,\infty )$ are related via (7.9). We first note that *f* belongs to $H^1_{\mathrm {loc}}[0,\infty )$ if and only if so does *g*. In this case, for a given function $h_\mathrm {s}$ in $H^1_{\mathrm {c}}[0,\infty )$, a substitution yields

$$\begin{aligned} \int _0^\infty f'(t)h_\mathrm {s}'(t)dt&= \int _0^\infty \biggl (g'(\mathrm {s}(t))+\frac{g(\mathrm {s}(t))}{2}\biggr ) \biggl (h'(\mathrm {s}(t)) + \frac{h(\mathrm {s}(t))}{2}\biggr ) \mathrm {s}'(t) dt \\&= \int _0^\infty g'(x)h'(x)dx + \frac{1}{4} \int _0^\infty g(x)h(x)dx - \frac{1}{2} g(0)h(0), \end{aligned}$$

where the functions *h* and $h_\mathrm {s}$ in $H^1_{\mathrm {c}}[0,\infty )$ are related by

$$\begin{aligned} h(x)&= h_\mathrm {s}(\mathrm {s}^{-1}(x))\mathrm {e}^{-\frac{x}{2}}, \quad x\in [0,\infty ),&h_\mathrm {s}(t)&= h(\mathrm {s}(t))\sqrt{1+t}, \quad t\in [0,\infty ). \end{aligned}$$

Furthermore, one computes that

$$\begin{aligned} \int _0^\infty {\mathsf {w}}_\mathrm {s}(t) (fh_\mathrm {s})'(t)dt&= - \int _0^\infty u(x)g(x) h(x)dx - \int _0^\infty u'(x)(gh)'(x)dx \end{aligned}$$

as well as

$$\begin{aligned} \int _{[0,\infty )} fh_\mathrm {s}\, d\upsilon _\mathrm {s}&= \int _{[0,\infty )} g(\mathrm {s}(t))h(\mathrm {s}(t)) d\upsilon \circ \mathrm {s}(t) = \int _{[0,\infty )} gh\, d\upsilon . \end{aligned}$$

With the help of these identities, the claim follows readily from the very definition of the respective solutions. $\square$

In conjunction with [20, Lemma 4.2] and a simple substitution, this relation readily provides the following result.

### Corollary 7.2

If *z* belongs to ${\mathbb {C}}\backslash {{\mathbb {R}}}$, then there is an (up to scalar multiples) unique non-trivial solution $\psi$ of the differential equation (7.2) such that $\psi$ lies in $H^1[0,\infty )$ and $L^2([0,\infty );\upsilon )$.

### Proof

Let us suppose that two functions *f* and *g* in $H^1_{\mathrm {loc}}[0,\infty )$ are related via (7.9). Then the function *f* lies in ${\dot{H}}^1[0,\infty )$ if and only if the function *g* lies in $H^1[0,\infty )$. In fact, if *f* lies in ${\dot{H}}^1[0,\infty )$, then a substitution shows that

$$\begin{aligned} \int _0^\infty |f'(t)|^2 dt = \int _0^\infty \Bigl | g'(x) + \frac{1}{2}g(x)\Bigr |^2 dx < \infty . \end{aligned}$$

Now upon noting that for $R>0$ we have

$$\begin{aligned} \int _0^R \Bigl | g'(x) + \frac{1}{2}g(x)\Bigr |^2 dx = \int _0^R |g'(x)|^2 dx + \frac{1}{4} \int _0^R |g(x)|^2 dx + \frac{|g(R)|^2 - |g(0)|^2}{2}, \end{aligned}$$

we see that *g* lies in $H^1[0,\infty )$ since it is bounded, which follows because *f* grows at most like a square root. The converse implication is straightforward. Moreover, we easily see that the function *f* lies in $L^2([0,\infty );\upsilon _\mathrm {s})$ if and only if the function *g* lies in $L^2([0,\infty );\upsilon )$. Now the claim follows readily from [20, Lemma 4.2]. $\square$

This result allows us to define the Weyl–Titchmarsh function *m* associated with the spectral problem (7.2) by

7.10 $$\begin{aligned} m(z) = \frac{\psi '(z,0-)}{z\psi (z,0)},\quad z\in {\mathbb {C}}\backslash {{\mathbb {R}}}, \end{aligned}$$

where $\psi (z,\cdot \,)$ is a non-trivial solution of the differential equation (7.2) which lies in $H^1[0,\infty )$ and $L^2([0,\infty );\upsilon )$. In view of Lemma 7.1, we readily compute that

7.11 $$\begin{aligned} m(z) = m_\mathrm {s}(z) - \frac{1}{2z}, \quad z\in {\mathbb {C}}\backslash {{\mathbb {R}}}, \end{aligned}$$

where $m_\mathrm {s}$ is the Weyl–Titchmarsh function of the corresponding generalized indefinite string $(\infty ,\omega _\mathrm {s},\upsilon _\mathrm {s})$. In particular, we see that *m* is a Herglotz–Nevanlinna function and the Borel measure $\mu$ in the corresponding integral representation differs from the one for the generalized indefinite string only by a point mass at zero. The measure $\mu$ is a spectral measure for a self-adjoint realization $\mathrm {T}_0$ of the spectral problem (7.2) in the Hilbert space

7.12 $$\begin{aligned} {\mathcal {H}}_0 = H^1[0,\infty )\times L^2([0,\infty );\upsilon ) \end{aligned}$$

equipped with the scalar product

7.13 $$\begin{aligned} \begin{aligned} \langle f , g \rangle _{{\mathcal {H}}_0}&= \int _0^\infty f_1'(x)g_1'(x)^*dx + \frac{1}{4}\int _0^{\infty } f_1(x)g_1(x)^*dx \\&\quad + \int _{[0,\infty )} f_2(x)g_2(x)^*d\upsilon (x), \quad f,\, g\in {\mathcal {H}}_0, \end{aligned}\end{aligned}$$

defined by saying that a pair $(f,g)\in {\mathcal {H}}_0\times {\mathcal {H}}_0$ belongs to $\mathrm {T}_0$ if and only if

7.14 $$\begin{aligned} -f_1'' + \frac{1}{4} f_1&= \omega g_1 + \upsilon g_2,&\upsilon f_2 = \upsilon g_1, \end{aligned}$$

and $g_1$ satisfies the Dirichlet boundary condition $g_1(0)=0$ at zero; compare [3, 16] and [21, Subsection 4.1] in particular. Since this establishes an immediate connection between the spectral properties of $\mathrm {T}_0$ and the corresponding generalized indefinite string $(\infty ,\omega _\mathrm {s},\upsilon _\mathrm {s})$, we may now invoke Theorem 3.2.

### Theorem 7.3

If the function $u-1$ belongs to $H^1[0,\infty )$ and the measure $\upsilon$ is finite, then the essential spectrum of $\mathrm {T}_0$ coincides with the interval $[1/4,\infty )$ and the absolutely continuous spectrum of $\mathrm {T}_0$ is essentially supported on $[1/4,\infty )$.

### Proof

Under these assumptions, we readily see that the coefficients of the corresponding generalized indefinite string $(\infty ,\omega _\mathrm {s},\upsilon _\mathrm {s})$ satisfy

$$\begin{aligned} \int _0^\infty \Bigl |{\mathsf {w}}_\mathrm {s}(t) + 1 -u(0) - \frac{t}{1+t} \Bigr |^2 t\, dt&\le \int _0^\infty |u'(\mathrm {s}(t))+ u(\mathrm {s}(t))-1|^2 \frac{1}{1+t} dt \\&= \int _0^\infty |u'(x)+ u(x)-1|^2 dx < \infty , \end{aligned}$$

upon performing a substitution, as well as

$$\begin{aligned} \int _{[0,\infty )} t\, d\upsilon _\mathrm {s}(t) \le \int _{[0,\infty )} d\upsilon \circ \mathrm {s}= \int _{[0,\infty )} d\upsilon <\infty . \end{aligned}$$

Now the claim follows from Theorem 3.2 with $c=u(0)-1$, $\alpha =1/4$ and $\eta =1$.

$$\square $$

Of course, it is also desirable to consider the spectral problem for (7.2) on the whole real line. In this case, a linear relation $\mathrm {T}$ in the Hilbert space

7.15 $$\begin{aligned} {\mathcal {H}}= H^1({{\mathbb {R}}})\times L^2({{\mathbb {R}}};\upsilon ) \end{aligned}$$

equipped with the scalar product

7.16 $$\begin{aligned} \begin{aligned} \langle f , g \rangle _{{\mathcal {H}}}&= \int _{{\mathbb {R}}}f_1'(x)g_1'(x)^*dx + \frac{1}{4}\int _{{\mathbb {R}}}f_1(x)g_1(x)^*dx \\&\quad + \int _{{{\mathbb {R}}}} f_2(x)g_2(x)^*d\upsilon (x), \quad f,\, g\in {\mathcal {H}}, \end{aligned}\end{aligned}$$

is given by defining that a pair $(f,g)\in {\mathcal {H}}\times {\mathcal {H}}$ belongs to $\mathrm {T}$ if and only if

7.17 $$\begin{aligned} -f_1'' + \frac{1}{4} f_1&= \omega g_1 + \upsilon g_2,&\upsilon f_2 = \upsilon g_1. \end{aligned}$$

This maximally defined linear relation $\mathrm {T}$ is self-adjoint and, as shown in the proof of [21, Lemma 5.2], it is a finite rank perturbation of two half-line problems, which allows us to use the Weyl and Rosenblum–Kato theorems about stability of the essential and absolutely continuous spectra (see [32, Chapter 10 §4] for example) to obtain the following result.

### Theorem 7.4

Let *u* be a real-valued function on ${{\mathbb {R}}}$ such that $u-1$ belongs to $H^1({{\mathbb {R}}})$ and let $\upsilon$ be a non-negative finite Borel measure on ${{\mathbb {R}}}$. Then the essential spectrum of $\mathrm {T}$ coincides with $[1/4,\infty )$ and the absolutely continuous spectrum of $\mathrm {T}$ is of multiplicity two and coincides with $[1/4,\infty )$.

## Schrödinger Operators with $\delta '$-Interactions

Our main theorems also apply to Schrödinger operators with $\delta '$-interactions. To this end, let $\nu$ be a real-valued Borel measure on $[0,\infty )$ which is singular with respect to the Lebesgue measure. For the sake of simplicity, we shall also assume that $\nu$ does not have a point mass at zero. The Borel measure $\omega$ on $[0,\infty )$ is then defined as the sum of the measure $\nu$ and the Lebesgue measure, that is,

8.1 $$\begin{aligned} \omega (B) = \nu (B) + \int _Bdx \end{aligned}$$

for every Borel set $B\subseteq [0,\infty )$. We consider the operator $H_\nu$ in the Hilbert space $L^2[0,\infty )$ associated with the differential expression

8.2 $$\begin{aligned} \tau _\nu = -\frac{d}{dx}\frac{d}{d\omega (x)} \end{aligned}$$

and subject to a Neumann boundary condition at zero. The operator $H_\nu$ can be viewed as a *Hamiltonian with* $\delta '$*-interactions*. Namely, if $\nu$ is a discrete measure such that

8.3 $$\begin{aligned} \nu = \sum _{s\in X}\beta (s) \delta _s, \end{aligned}$$

where *X* is a discrete subset of $[0,\infty )$, $\beta$ is a real-valued function on *X* and $\delta _s$ is the unit Dirac measure centred at *s*, then the differential expression $\tau _\nu$ can be formally written as (see [23, Example 2.2])

8.4 $$\begin{aligned} -\frac{d^2}{dx^2} + \sum _{s\in X}\beta (s)\langle \,\cdot \,,\delta _s'\rangle \delta _s', \end{aligned}$$

which is the Hamiltonian with $\delta '$-interactions on *X* of strength $\beta$ (see [1, 35, 36]). It is known (see [23] and [24]) that under the above assumption on $\nu$, the operator $H_\nu$ is self-adjoint in $L^2[0,\infty )$. The spectral properties of $H_\nu$ turn out to be closely connected with those of the generalized indefinite string $S_\nu = (\infty ,\omega ,0)$.

### Lemma 8.1

The operator $H_\nu$ is unitarily equivalent to $S_\nu$.

### Proof

Since $\tau _\nu$ is in the limit point case at $\infty$ (see [23]), for every $z\in {\mathbb {C}}\backslash {{\mathbb {R}}}$ there is an (up to scalar multiples) unique non-trivial solution $\psi _\nu (z,\cdot \,)$ to $\tau _\nu y = zy$ such that $\psi _\nu (z,\cdot \,)$ lies in $L^2[0,\infty )$. Recall that the Weyl–Titchmarsh function $m_\nu$ of $H_\nu$ is then given by

$$\begin{aligned} m_\nu (z) = - \frac{\psi _\nu (z,0)}{\psi _\nu ^{[1]}(z,0)},\quad z\in {\mathbb {C}}\backslash {{\mathbb {R}}}, \end{aligned}$$

where the function in the denominator is the quasi-derivative

$$\begin{aligned} \psi _\nu ^{[1]}(z,\cdot \,) = \frac{d\psi _\nu (z,\cdot \,)}{d\omega }. \end{aligned}$$

It is known that $m_\nu$ is a Herglotz–Nevanlinna function and the Borel measure in the corresponding integral representation is a spectral measure for the operator $H_\nu$. Now for every function $h\in H^1_{\mathrm {c}}[0,\infty )$ we compute

$$\begin{aligned} \int _0^\infty \psi _\nu ^{{[1]}\prime }(z,x)h'(x)dx&= -z\int _0^\infty \psi _\nu (z,x)h'(x)dx \\&= z \psi _\nu (z,0) h(0) + z\int _0^\infty \psi _\nu ^{[1]}(z,x) h(x) d\omega (x), \end{aligned}$$

where we used the fact that $\psi _\nu ^{{[1]}\prime } = -z\psi _\nu$ almost everywhere as well as an integration by parts (use [6, Exercise 5.8.112] or [28, Theorem 21.67] for example). This shows that the quasi-derivative is a solution of the differential equation

$$\begin{aligned} -f'' = z\, \omega f \end{aligned}$$

with $\psi _\nu ^{{[1]}\prime }(z,0-)=-z\psi _\nu (z,0)$. Since it furthermore lies in ${\dot{H}}^1[0,\infty )$, the Weyl–Titchmarsh function *m* of the generalized indefinite string $S_\nu$ is given by

$$\begin{aligned} m(z) = \frac{\psi _\nu ^{{[1]}\prime }(z,0-)}{z\psi _\nu ^{[1]}(z,0)} = \frac{-z\psi _\nu (z,0)}{z\psi _\nu ^{[1]}(z,0)} = m_\nu (z), \quad z\in {\mathbb {C}}\backslash {{\mathbb {R}}}. \end{aligned}$$

Thus, the corresponding spectral measures coincide and hence the claim follows. $\square$

Recall that the normalized anti-derivative ${\mathsf {v}}$ of the measure $\nu$ is given by

8.5 $$\begin{aligned} {\mathsf {v}}(x)=\int _{[0,x)}d\nu \end{aligned}$$

for almost all $x\in [0,\infty )$. The connection established in Lemma 8.1 now allows us to apply Theorem 3.1.

### Theorem 8.2

Suppose that

8.6 $$\begin{aligned} \int _{0}^\infty |{\mathsf {v}}(x) - c|^2dx<\infty \end{aligned}$$

for a real constant *c*, where ${\mathsf {v}}$ is the normalized anti-derivative of $\nu$. Then the essential spectrum of the Hamiltonian $H_\nu$ coincides with the interval $[0,\infty )$ and the absolutely continuous spectrum of $H_\nu$ is essentially supported on $[0,\infty )$.

### Proof

This is a simple consequence of Lemma 8.1 and Theorem 3.1. $\square$

### Remark 8.3

In conclusion, let us mention that one can say more about spectral properties of the Hamiltonian $H_\nu$ and about its negative spectrum in particular. Namely, it is possible to show that the negative spectrum consists of simple eigenvalues which may only accumulating at $-\infty$. More specifically, they satisfy the following Lieb–Thirring-type bound

8.7 $$\begin{aligned} \sum _{\lambda \in \sigma (H_\nu )\cap (-\infty ,0)} \frac{1}{|\lambda |^{3/2}} \le \frac{3}{4}\int _{0}^\infty |{\mathsf {v}}(x) - {\mathsf {v}}_0|^2dx. \end{aligned}$$

However, we postpone the discussion of Lieb–Thirring-type inequalities to a forthcoming publication.

## Footnotes

1 In the following, we will always take the branch of the square root $\sqrt{\cdot }$ with cut along the positive semi-axis $[0,\infty )$ defined by $\sqrt{z}=\sqrt{|z|}\mathrm {e}^{\mathrm {i}\arg (z)/2}$ with $\arg (z)\in [0,2\pi )$.
